# The extent of the neurocognitive impairment in elderly survivors of war suffering from PTSD: meta-analysis and literature review

**DOI:** 10.3934/Neuroscience.2021003

**Published:** 2020-11-25

**Authors:** Yasir Rehman, Cindy Zhang, Haolin Ye, Lionel Fernandes, Mathieu Marek, Andrada Cretu, William Parkinson

**Affiliations:** 1 Health Research Methodology, McMaster University, Hamilton, ON, Canada; 2 Faculty of Health Sciences, McMaster University, Hamilton ON, Canada; 3 Faculty of Life Sciences, McMaster University, Hamilton ON, Canada; 4 School of Rehabilitation Science, McMaster University, Hamilton ON, Canada

**Keywords:** PTSD, survivors of war, elderly, neurocognitive function, meta-analysis

## Abstract

**Objectives:**

We performed a meta-analysis and systematic review on elderly survivors of war suffering from PTSD to estimate the variability in their cognitive impairment based on individual neuropsychological tests.

**Methods:**

We included case control studies that explored the association of cognitive deficits in elderly PTSD civilian survivor of wars (age >60 years), using MEDLINE, Embase and PsycINFO from the inception to January 2018. We compared the cognitive performances in three comparisons i) PTSD+ vs. PTSD− civilian survivors of war; ii) PTSD+ vs. Control and iii) PTSD− vs. Control. The risk of bias was assessed using the Newcastle-Ottawa Scale for case-control studies.

**Results:**

Out of 2939 titles and abstracts, 13 studies were eligible for data extraction. As compared to PTSD− civilian survivors of war, PTSD+ civilian survivors of war demonstrated significant deficits on TMT-A, TMT-B, Digit span backward, explicit memory low pair associate, CVLT recognition, WAIS-verbal and non-verbal tests. As compared to health controls, PTSD+ survivors demonstrated significantly lower performance on explicit memory low pair and high associate, RAVLT immediate and delayed recall, CVLT delayed and short cued recall. Performance on the neuropsychological test between PTSD− survivors of war and controls was not significant for all tests.

**Conclusion:**

The pattern suggests that PTSD+ survivors of war had poorer performance in tasks requiring processing speed, executive function, attention, working memory and learning. The magnitude of the cognitive deficits in our pooled analysis was small to moderate depending on the neuropsychological test. Most of our pooled analysis suffered from a high risk of bias, which lowered the confidence in our results.

## 1. Introduction

Elderly patients with PTSD have been found to have greater impairments in their cognitive performance [1]–[3], which hinders their ability to cope and rehabilitate [4],[5]. Prevalence of PTSD in survivors of war including war veterans and civilian population has been reported from 17% to 30% [6],[7] and symptoms may appear two to four decades after the initial war-related trauma [8]–[10]. Elderly veterans and survivors of war have a higher prevalence of impairment in cognitive functioning [11]–[18] and comorbid ailments such as depression, head injuries and medical comorbidities [6],[7]. They also experience a poor quality of life and impairments in their social lives [7],[18]–[21]. The aging, cognitive impairments in elderly survivors of war and negative reaction such as intrusive memories, thought processing, physical and emotional changes further complicate the distress and reduce treatment responses [22]–[24].

Cook et al. [18] reported that cognitive impairment in elderly PTSD patients does not differ significantly from cognitive impairment in dementia patients without PTSD. By understanding the extent of cognitive impairments in elder PTSD patients, we can facilitate understanding to improve treatment modalities, functional outcomes, and health outcomes. The goal of our study was to systematically explore the extent of the neurocognitive domain in elderly survivors of war in five major domains such as i) learning and memory; ii) Attention; iii) executive functions; iv). language and v). visuospatial processing [25],[26]. As in elderly patients, cognitive deficits can be confounded due to aging; therefore, we aim to explore the quality of evidence in outcomes across different comparisons i) PTSD+ vs. PTSD−; ii) PTSD+ vs. Healthy Controls and iii) PTSD− survivors of war vs. Healthy Controls.

Previous studies inconsistently reported major neurocognitive dysfunction in elderly survivors of wars suffering from PTSD, such as autobiographical memory [23],[27], attention [8], verbal learning and memory [28],[29], executive function and information processing speed [1],[30]–[32]. Previous studies mostly focused on veterans and omitted civilians with wartime traumatic events [33]–[35]. As elderly survivors of war suffer from other ailments, previous reviews included studies duplicate studies on the same populations [11] or with heterogeneous populations [11],[12] with mixed trauma and medical conditions which make it difficult to determine the independent association of PTSD with neurocognitive deficits. Previous reviews [11],[12] also did not determine the quality of evidence in the outcomes. Studies exploring the overall extent of neurocognitive deficits in survivors of war have rarely been reported. Our goal was to explore the extent of performance on neuropsychological tests in elderly PTSD+ survivors of wars. Also, to our knowledge, no previous published meta-analyses on this topic have employed the GRADE criteria (Grading of Recommendations, Assessment, Development and Evaluation) to calculate the quality of evidence.

## 2. Methods

A systematic review and meta-analysis were conducted according to the Preferred Reporting Items for Systematic Review and Meta-analysis (PRISMA) guidelines [36]. The search strategy was developed in OVID Medline, PsycINFO, and EMBASE from the inception to January 2018 (See the appendix for the search strategy). We also looked for the bibliographic references for the recently published systematic reviews (see PRISMA flowchart in the appendix). Literature screening for title and abstract, full text, the risk of bias and data extraction were done in duplicates and independently. Our protocol was registered on Prospero (CRD42018090134). For this review, we included studies that explored the neurocognitive deficits in outpatient elderly survivors of wars suffering from PTSD with validated neuropsychological tools. Studies compared PTSD+ survivors of war with healthy control or PTSD− the survivor of war and reported effect size were in mean and standard deviation. Studies were excluded if enrolled participants with traumatic brain injury, neurodegenerative/neuroinflammatory diseases, psychosis, or PTSD due to other conditions such as motor vehicle accidents, rape, or domestic violence. Studies were also excluded if they analyzed brain functional imaging in PTSD patients without reporting neuropsychological tests scores. We restricted the inclusion of eligible studies to the English language only.

### 2.1. Important definitions

Elderly age: As the elderly population is defined variably in different cultures, which may range from 60 to above 65 years. For this review, we included studies enrolling patients with average age of 60 or above [5],[37],[38].

PTSD+ Survivors of war: PTSD status was determined if the study employed DSM criteria, CAPS criteria or explicitly determined by clinicians. For this review, combat veterans, prisoners of wars, and/or civilians, who were exposed to war trauma were considered survivors of war. For this review, combat veterans, prisoners of wars, and/or civilians, who were exposed to war trauma were considered survivors of war. The severity of PTSD was based on scores reported for Clinician-Administered PTSD Scale (CAPS) [39], Post-traumatic Stress Diagnostic Scale (PDS) [40], PTSD symptoms scale (PSS tests) [41],[42] scales. The severity of CAPS is categorized as: 0–19 = asymptomatic/few symptoms; 20–39 = mild PTSD/subthreshold; 40–59 = moderate PTSD/threshold, 60–79 = severe PTSD symptomatology; >80 = extreme PTSD symptomatology. The severity on PDS is categorized as 0 no rating, 1–10 mild, 11–20 moderate, 21–35 moderate to severe, and >36 severe. Hart et al. [34] did not report the PTSD severity, whereas Golier et al. [43]–[45] and Yehuda [46],[47] reported individual scores for the intrusion, avoidance and hyperarousal. As these studies used same population as Freeman et al. [48] and Yehuda et al. [49] respectively, we assumed the PTSD severity approximately the same as reported in the latter.

Comparison groups were either individual exposed to war-related trauma or healthy controls but were not diagnosed with PTSD. Our rationale to compare the cognitive impairment in PTSD+ with PTSD− survivor of wars was based, as war trauma can be potentially associated with other ailments such as depression, medical comorbidities, which confound the association if comparison with only healthy control made.

Neurocognitive domains and neuropsychological tests: We focused on five major neurocognitive domains such as learning and memory, attention, executive functions, language, and visuospatial processing [25],[26]. We further captured data on the subdomain [50] of each cognitive function such as inhibition and flexibility are subdomains of executive functions and pooled them separately according to the neuropsychological tests [51],[52]. We included studies that used valid neuropsychological tests to measure cognitive functions. Common versions are listed in Table 1: Descriptive table of neuropsychological tests, and cognitive functions.

## 3. Risk of bias

The risk of bias was evaluated in the eligible studies using the Newcastle-Ottawa Quality Assessment scale for case-control studies [53]. Newcastle-Ottawa Scale (NOS) assesses the following three domains: the selection of study groups, comparability of study groups and ascertainment of exposure or outcome. For this analysis, case definition and control representations were rated if individuals were recruited through consecutive sampling, national databases and or veteran registries. If the source of participant recruitment was not clearly reported or if it were recruited with convenience sampling, the study was down rated. For the case comparison, we used two variables (age and premorbid IQ). If the study had a significant difference for age and/or premorbid IQ between case and control groups, the study was down rated. Outcome assessment (method of ascertainment) was determined downrated if the study did not employ validated neurocognitive test or if cognitive impairment was determined differently for case and control groups. Also, studies were down rated if the authors reported results selectively and did not report results for all outcomes in the method section.

## 4. Grade

The quality of evidence was determined with the Grading of Recommendations Assessment, Development, and Evaluation (GRADE) [54]. We will use GRADE to rate the evidence separately for each cognitive sub domain. Assessment of GRADE is based on 5 variables i) Risk of bias; ii) Heterogeneity; iii) precision; iv) publication bias and v) indirectness. The risk of bias was determined component by component and heterogeneity was explored with the visual inspection of forest plot and I^2^. The publication was assessed with the visual inspection of a funnel plot if we had 10 or more studies in a pooled analysis.

Subgroup analysis: we did not have enough studies to perform subgroup analysis described a priori in Prospero protocol.

**Table 1.** Descriptive table of neuropsychological tests, neurocognitive functions and authors.

Cognitive Test	Description	Cognitive Domain(s)	Studies Employing this Test (authors)
Trail Making Test A	records the time required to connect numbered dots spread randomly over a page	Information Processing Speed, Visual Scanning, Attention	Green 2016, Hart 2008, Jelinek 2013
Trail Making Test B	participants must alternate between ascending numeric and alphabetical characters in connecting the dots	Mental Flexibility, Executive functioning, Attention Shifting	Green 2016, Hart 2008, Jelinek 2013
WAIS—Digit Span Forward	participants are asked to immediately repeat longer strings of digits in the same order (Forward subtest)	Attention efficiency and capacity	Hart 2008, Jelinek 2013
Digit Span—Backward	Participants are asked to immediately repeat longer strings of digits in the reverse order (Backwards subtests)	Executive function dependent on working memory	Hart 2008, Jelinek 2013
Color Word Interference	While being timed, participants were asked to say the ink color that various words were written in	Inhibition of cognitive interference	Green 2016, Wittekind 2010
Pair Associate tests	Participants were given 6 pairs of related words (high) and 6 pairs of unrelated words (low) and were asked to recall immediately after tests and after 30 minutes of the test administration. Patients were shown one pair and were asked to recall the other word. For the implicit memory patients were asked to complete 48 three-letter word stems using “the first word that comes to mind”	Learning of complex information associated with Explicit Memory and implicit memory	Yehuda 2005, Yehuda 2006
RAVLT—Immediate and delayed Recall	A word list is read out and participants attempt to immediately recall as many as possible in repeated trials. A new list is read out and immediately after the trial is completed. In delayed recall, patients are asked to recall the information 20-30 minutes	Short-term and long-term verbal memory, rate of learning, memory retention, effects of interference on verbal leaning	Freeman 2006, Green 2016, Wessel 2002, Yehuda 2004, Yehuda 2006
CVLT—Short Cued and Delayed Cued Recall	List of nouns repeated aloud in the same order, from categories (fruit, clothing, etc.) followed by interference list. Participants must recall words in any order, when given categories (cued)	Short-term and long-term memory retrieval or recall in response to cues	Yehuda 2004, Yehuda 2005, Yehuda 2006
CVLT—Recognition Memory	Participants must recognize words in a list of 44 words with target and distracter words	Verbal memory and learning associated with the recognition memory associated with objects, naming	Freeman 2006, Hart 2008, Yehuda 2004
Digit Symbol—Processing Speed	Each number is assigned a symbol and the participant must write the corresponding symbol when given a list of numbers	Information Processing Speed, short-term memory	Hart 2008
WAIS—Vocabulary	12-word pairs: 6 pairs of mildly related words (high associate) and 6 pairs of unrelated words (low associate), which the participants viewed and were then asked to read and memorize for recall. They were then shown a single word from each pair and attempted to recall the other	Verbal Intelligence, working memory	Golier 2003, Yehuda 2004, Yehuda 2005, Yehuda 2007
WAIS nonverbal intelligence	Participants rearranged blocks by hand that had various color patterns on various sides to match an arbitrary pattern	short-term memory, mental manipulation, holding time, motor skill, Spatial Visualization	Yehuda 2004, Yehuda 2005, Yehuda 2007
Boston Naming Test	subjects are asked to name objects that were presented visually in two dimensional lines. Measured with correct number of names produced	Naming objects	Hart 2008
COWA test	Spontaneous production of words beginning with the same letter	Verbal fluency	Hart 2008
Corsi Block Tapping Test	Tapping on a sequence of up to nine blocks mimicking a researcher	Visuospatial memory	Jelnik 2013
Groningen Intelligence Test (GIT)	Patients were asked to produce words related to a category	Semantic fluency	Wessel 2002
Animal fluency	Patients were asked to produce words related to a category	Semantic fluency	Hart 2008
Symbol Digit Modalities Test	Patients were asked to write or say the correct number for each symbol that was shown earlier	Information processing speed, attention	Hart 2008
Autobiographical memory test	Patients were asked to recall in response to specific cues provided to them in a specific time; The cues could be emotionally positive or negative valence	Learning and memory	Wessel 2002
WMS—Logical memory	Participants were asked to recall the details and themes of the two passages read immediately and after 20–30 minutes	Learning and memory	Freeman 2006

## 5. Analysis and data pooling

We reported study characteristics narratively. As a neuropsychological test can be used to measure more than one cognitive function, we pooled our analysis according to the neuropsychological tests rather than neurocognitive functions. We further captured data on the subdomain of each cognitive function such as working memory, inhibition and mental flexibility are subdomains of executive functions and pooled them separately according to the neuropsychological tests. If a test had subcomponents such as Trails Making Test (TMT) has two components TMT-A and TMT-B we pooled each component separately [51],[52]. Also, if different tests seemed homogenous in measuring cognitive function, were also pooled for the analysis purposes. A similar approach to standardize the comparison was also adopted in Schuitevoerder et al. [12]. We performed a meta-analysis if a neuropsychological test was reported in two or more studies. We also extracted data on commonly used neuropsychological tests if reported in a single study; Wechsler Memory Scale (WMS) Logical memory, recall score, WMS-logical memory thematic score, WMS-digit symbol, symbol digit modalities, corsiblock tapping test, Controlled Oral Word Association Test (COWA), and semantic fluency.

Also, to explore, an association of cognitive deficits with PTSD, we performed 3 different comparisons: PTSD+ vs. PTSD−, PTSD+ vs. Healthy Controls, and PTSD− vs. Healthy Controls. We observed variability in measuring and final reporting of results in the three comparisons. After extracting data, we determined studies if pooling was feasible. In the case of duplicate studies or multiple studies using the same population, we extracted data with a larger sample size and study matched for important variables for the risk of bias components. Results for pooled analysis were reported in mean difference (MD) with 95% CI. We performed pooling with a fixed effect model (FEM) if we had two studies in a meta-analysis and with random effect model (REM) if we had three or more studies. Meta-analysis was performed using Review Manager Software 5.3.

## 6. Results

Out of 4598 title and abstracts, 13 studies were eligible for data extraction (Figure 1). The summary of the included studies and neuropsychological tests are given in tables 1 and 2 respectively. Three studies [34],[49],[55] were duplicate for Golier et al. [56], Freeman et al. [49] and Jelenik et al. [57] respectively; therefore, we reported data only for tests if it were not reported in previous studies. The median sample size for PTSD+ survivors of war, PTSD−survivors of war and control groups were 20, 16 and 19, respectively. The median age for PTSD+, PTSD− and healthy control groups were 69.7, 68.4, and 70.9, respectively. Except for four studies [33]–[35],[58]; all other studies included both men and women. Most studies enrolled patients who survived World War II, whereas two studies [34],[48] recruited survivors of Korean Wars and one study [58] enrolled survivors of Dutch or Dutch-Indonesian civilians including those who survived Japanese concentration camps. Golier et al. [43],[44], reported data on the same population but tests were different. All studies excluded patients with substance abuse except for one study [58]. The PTSD severity was within the moderate range, except for the two studies [55],[57] with participants in moderate to severe range. Except for Freeman et al. [48], no other study reported combat related stress in the participants.

**Table 2.** Study characteristics of included articles.

Authors	Population	Sample size			Female-Sex (%) Age in Mean (SD)			Exclusion Criteria			PTSD severity score	Psychological interventions
		PTSD+	PTSD−	Healthy Control	PTSD+	PTSD−	Healthy Control	Substance Abuse Disorder	Psychological Deficits	Brain Injury/ Neurological Deficits		
Freeman 2006	POW—WWII, Korean War	10	10	6	All male patients			Yes	Yes, except depression	Yes	CAPS = 53.3 (13.9)	No
					Age: 79.6 (3.2)	Age: 79.8 (2.8)	Age: 80.8 (3.5)					
Golier 2002	Holocaust Survivors	31	16	35	67.7%	68.8%	57.1%	Yes	Yes, except depression	Yes	CAPS = 64.9 (15.4)	Yes
					Age: 67.7 (5.6)	Age: 67.4 (5.8)	Age: 69.9 (6.6)					
Golier 2003	Holocaust Survivors	31	16	34	67.7%	68.8%	57.1%	Yes	Yes, except depression	Yes	CAPS = 68.8	Yes
					Age: 67.7 (5.6)	Age: 67.4 (5.8)	Age: 69.9 (6.6)					
Golier 2005	Holocaust Survivors	14	13	20	63.4%	53.84%	35%	Yes	Yes, except depression	Yes	CAPS = 64.9 (15.4)	Yes
					Age: 70.5 (5.6)	Age: 68.5 (7.3)	Age: 71.4 (6.4)					
Green 2016	Vietnam Veteran	55	33	NA	All male patients			Yes	Yes	Yes	CAPS = 46.5 (23.04)	NR
					Age: 61 (4.3)	Age: 66.1(7.5)						
Hart 2008	POW, WW II, Korean War	7	11	NA	All male patients			Yes	Yes, except depression	Yes	CAPS = 53.3 (13.9)	No
					Age: 80.9 (2.51)	Age: 80 (2.2)	NA					
Jelinek 2013	WW II	20	24	11	70%	62.5%	63.3%	Yes	Yes, except depression	Yes	PDS = 21.05 (7.20)	NR
					Age: 70.95 (2.51)	Age: 70.88 (1.78)	Age: 72.27 (2.87)					
Wessel 2002	WW II, Indonesian War	25	NA	15	60%	0%	40%	No	No	Not Clear	PSS = 24.5 (10.9)	NR
					Age: 60.3 (3.8)	NA	Age: 62.3 (4.3)					
Wittekind 2010	WW II	22	24	11	68.2%	62.5%	63.60%	Yes	Yes, except depression	Yes	PDS = 20.05 (7.61)	NR
					Age: 71 (2.39)	Age: 70.88 (1.78)	Age: 72.27 (2.87)					
Yehuda 2004	Holocaust Survivors	36	26	40	67.74%	65.4%	55%	Yes	Yes, except depression	Yes	CAPS = 64.9 (15.4)	PTSD+ = 19% PTSD− = 23.1%
					Age: 69.2 (5.6)	Age: 68.4 (6.4)	Age: 70.4 (6.8)					
Yehuda 2005	Holocaust Survivors	19	16	28	63.12%	56.25%	53%	Yes	Yes, except depression	Yes	CAPS = 64.9 (15.4)	Yes
					Age: 69.7 (5)	Age: 70.2 (6.9)	Age: 73 (6.3)					
Yehuda 2006	Holocaust Survivors	14	13	19	All male patients		57.9%	Yes	Yes, except depression	Yes	CAPS = 64.9 (15.4)	No
					Age: 72.9 (6)	Age: 72.7 (6.3)	Age: 76.4 (6.8)					
Yehuda 2007	Holocaust Survivors	17	16	NA	All male patients			Yes	Yes, except depression	Yes	CAPS = 45.5 (25.6)	No
					Age: 60.6 (7)	Age: 65.1 (9.9)						

PDS = Post-traumatic Stress Diagnostic Scale, CAPS = Clinician-Administered PTSD Scale, PSS = PTSD symptoms scale, WW II = World war II, POW = Prisoner of War

### 6.1. Risk of bias and quality of evidence

The risk of bias of the eligible study is given in table 3. Except for Wessel [58], all studies recruited patients with consecutive sampling. For a case comparison, significant difference between premorbid IQ was reported in Freeman 2006 [48], Golier 2002 [43], Golier 2003 [44], Golier 2005 [45], Green 2016 [33], Hart 2008 [34], Wessel 2002 [58], and Yehuda 2005 [46], Golier 2002 [43], Golier 2003 [44] and Golier 2005 [45], were downrated for poor response rate.

**Table 3.**
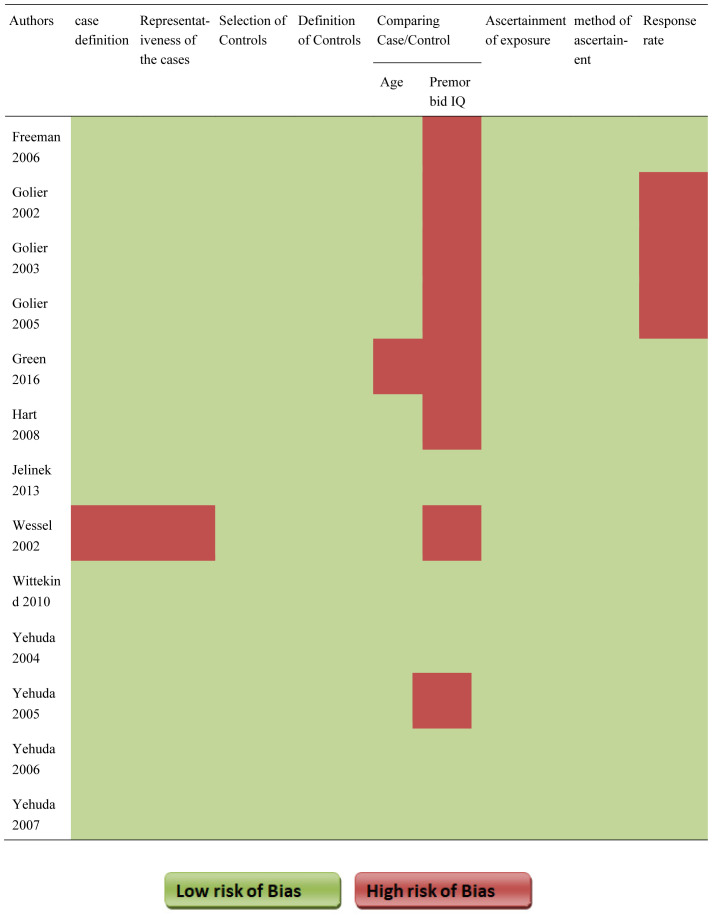
Risk of bias of the included studies (Newcastle-Ottawa scale)

### 6.2. Pooled analysis

Table 4 shows the meta-analysis for three comparisons PTSD+ vs. PTSD−, PTSD+ vs. Health control and PTSD− vs. Healthy control. Results are reported in according to the neuropsychological tests and the neurocognitive functions (Figures 2–9). The quality of evidence is reported in table 5.

### 6.3. PTSD+ vs. PTSD− survivors of war

Among the significant tests, moderate quality evidence was reported for Trail Making Test-A (TMT-A) (−6.05 [−11.04, −1.06]; *p* = 0.02; I^2^ = 28%); TMT-B (−25.80 [−43.70, −7.89]; *p* = 0.005; I^2^ = 46%]; Digit Span Backward (−0.82 [−1.63, −0.01]; *p* = 0.05; I^2^ = 0%); Stroop Color Word Interference (−8.35 [−16.50, −0.20]; *p* = 0.04; I^2^ = 0%); California Verbal Learning Test (CVLT)—Recognition Memory (−2.43 [−4.56, −0.30]; *p* = 0.03; I^2^ = 0%); CVLT—Delayed Cued Recall (−1.12 [−2.17, −0.07]; *p* = 0.04; I^2^ = 0%), Wechsler Adult Intelligence Scale (WAIS)—verbal intelligence (−2.64 [−3.38, −1.90]; *p* < 0.00001; I^2^ = 0%) and WAIS—nonverbal intelligence (−1.55 [−2.55, −0.54]; *p* = 0.002; I^2^ = 0%). Low quality evidence reported significant association on Explicit Memory—Low Pair Associate (−1.64 [−2.65, −0.63]; *p* = 0.001; I^2^ = 70%).

Among non-significant neuropsychological tests were Explicit Memory—High Pair Associate; Implicit Memory—High Pair Associate; Implicit Memory—Low Pair Associate; Rye Adult learning test (RAVLT)—Immediate Recall RAVLT-Delayed Recall; CVLT—Short Cued Recall; Free Recall—Delayed and Digit Span Forward.

### 6.4. PTSD+ vs. healthy control

Among the significant tests, moderate quality evidence was reported for Explicit Memory—High Pair Associate (−1.48 [−2.02, −0.94]; *p* < 0.00001; I^2^ = 0%); Explicit Memory—Low Pair Associate (−1.61 [−2.51, −0.71]; *p* = 0.0005; I^2^ = 87%); RAVLT—Immediate Recall (−1.08 [−1.90, −0.26]; *p* = 0.01; I^2^ = 70%); Free Recall—Delayed (−2.23 [−3.38, −1.09]; *p* = 0.0001; I^2^ = 0%); and CVLT—Recognition Memory [−4.10 [−6.67, −1.42]; *p* = 0.003; I^2^ = 0%]. Among the significant tests, high quality evidence was reported for CVLT Delayed Recall (−1.82 [−2.95, −0.69]; *p* = 0.002; I^2^ = 0%); CVLT—Short Cued Recall (−1.98 [−3.14, −0.81]; *p* = 0.0009; I^2^ = 50%); and CVLT—Delayed Cued Recall (−1.24 [−2.25, −0.23]; *p* = 0.02; I^2^ = 0%). Finally, low quality evidence was reported for the following significant test: WAIS—verbal intelligence (−3.46 [−4.29, −2.63]; *p* = 0.00001; I^2^ = 0%) and WAIS—non-verbal intelligence (−1.73 [−2.84, −0.62]; *p* = 0.002; I^2^ = 0%).

Among the non-significant tests were Implicit Memory—High Pair Associate; Implicit Memory—Low Pair Associate; and RAVLT—Delayed Recall.

### 6.5. PTSD− vs. healthy outcomes

In this comparison, none of the cognitive domains shows a significant association with the PTSD− survivor of war and healthy controls.

Summary of the neuropsychological tests that were reported by single studies that were not poolable is reported in table 6. PTSD+ survivor of war performed significantly poor on information process speed and autobiographical memory than the PTSD− survivors of war and healthy controls.

## 7. Discussion

Our aim was to systematically explore the association of neurocognitive impairments in elderly survivors of war suffering from PTSD. Our review identified elderly survivors of war with PTSD exhibited neurocognitive deficits on the neuropsychological tests requiring complex functions such as attention, information processing speed, executive functioning, learning and memory and intelligence tests. Attention and information processing showed elderly survivors of war with PTSD exhibited significant impairment as compared to elderly survivors of war who did not have PTSD. Attention measured on the digit span forward was not significant. Performance on tests measuring executive function was consistently reported significantly poor in elderly survivors of war than the non-PTSD survivors of war. The effect size was reported on the performance on TMT-B (MD = −25.80) and Stroop color word inhibition (MD = −8.35). We noted non-consistent association of neurocognitive deficits with test measuring learning and memory. As compared to PTSD− survivors of war, the elderly PTSD+ survivors of war exhibited poor performances on tests that measured delayed recall, information retrieval or on complex tasks such as low pair associates for explicit memory.

**Table 4.** Pooled analysis.

Neurocognitive function	Cognitive test/ subcomponent of cognitive test	PTSD+ vs. PTSD− survivor of wars		PTSD+ vs. Health Control		PTSD− vs. Health Control	
		Sample size	MD (95% CI)	Sample size	MD (95% CI)	Sample size	MD (95% CI)
Attention and Information processing speed	TMT-A	150	−6.05 [−11.04, −1.06]	NA	NA	NA	NA
	WAIS—Digit span Forward	62	−0.07 [−1.24, 1.09]	NA	NA	NA	NA
Executive function	TMT-B	150	−25.80 [−43.70, −7.89]	NA	NA	NA	NA
	WAIS—Digit span Backward	62	−0.82 [−1.63, −0.01].	NA	NA	NA	NA
	Stroop color word interference	134	−8.35 [−16.50, −0.20].	NA	NA	NA	NA
Learning and memory	Explicit Memory—High Pair Associate	74	−0.61 [−1.25, 0.03]	99	−1.48 [−2.02, −0.94].	83	−0.39 [−1.24, 0.45]
	Explicit Memory—Low Pair Associate	74	−1.64 [−2.65, −0.63]	99	−1.61 [−2.51, −0.71]	83	−0.29 [−1.23, 0.65,]
	Implicit Memory—High Pair Associate	62	−0.40 [−1.78, 0.99]	80	−0.05 [−1.35, 1.24]	76	−0.43 [−2.01, 1.14]
	Implicit Memory—Low Pair Associate	62	−0.90 [−2.58, 0.78]	80	−0.86 [−2.41, 0.70]	76	−0.03 [−1.72, 1.66]
	RAVLT—Immediate Recall	82	−0.77 [−2.04, 0.50]	132	−1.08 [−1.90, −0.26]	82	−0.96 [−2.24, 0.32]
Learning and memory	RAVLT—Delayed Recall	197	−0.98 [−1.80, −0.16]	165	−1.74 [−2.74, −0.73]	114	−0.46 [−1.81, 0.90]
	CVLT—Short Cued Recall	89	−1.04 [−2.36, 0.28].	109	−1.98 [−3.14, −0.81]	98	−0.71 [−1.89, 0.48]
	CVLT—Delayed Cued Recall	89	−1.12 [−2.17, −0.07]	109	−1.24 [−2.25, −0.23]	98	−0.12 [−0.94, 0.70]
	CVLT—Recognition Memory	100	−2.43 [−4.56, −0.30].	92	−4.10 [−6.77, −1.43]	83	−0.09 [−0.93, 0.75]
Verbal comprehension and Intelligence	WAIS—Verbal IQ	157	−2.64 [−3.38, −1.90]	188	−3.46 [−4.29, −2.63]	160	−0.73 [−1.55, 0.10]
Performance intelligence	WAIS—Non-Verbal IQ	95	−1.26 [−2.44, −0.08]	156	−1.46 [−2.46, −0.47]	110	−0.13 [−1.03, 0.78]

TMT (Trail making Test); WAIS (Wechsler Adult Intelligence Scale); RAVLT (Rey Auditory Verbal Learning Test); CVLT (California Verbal Learning Test); IQ (Intelligence quotient)

**Table 5.** GRADE quality of evidence.

Neurocognitive Domain	Neuropsychological Tests	Risk of bias	Heterogeneity	Precision	Indirectness	Publication bias	Final decision
Attention and Information processing speed	TMT-A	High risk	Low Risk	Low Risk	Not detected	Not assessed	0 + + + (moderate)
	WAIS—Digit span Forward	Low Risk	Low Risk	High Risk	Not detected	Not assessed	0 + + + (moderate)
Executive function	TMT-B	High risk	Low Risk	Low Risk	Not detected	Not assessed	0 + + + (moderate)
	Digit Span Backward	Low Risk	Low Risk	Low Risk	Not detected	Not assessed	0 + + + (moderate)
	Color Word Interference—Inhibition	High risk	Low Risk	Low Risk	Not detected	Not assessed	0 + + + (moderate)
Learning and memory	RAVLT—Immediate Recall	High risk	Low Risk	High Risk	Not detected	Not assessed	0 0 + + (Low)
	RAVLT—Delayed Recall	High risk	Low Risk	High Risk	Not detected	Not assessed	0 0 + + (Low)
	Explicit Memory—High Pair Associate	High risk	Low Risk	High Risk	Not detected	Not assessed	0 0 + + (Low)
	Explicit Memory—Low Pair Associate	High risk	Low Risk	Low Risk	Not detected	Not assessed	0 + + + (moderate)
	Implicit Memory—High Pair Associate	High risk	Low Risk	High Risk	Not detected	Not assessed	0 0 + + (Low)
	Implicit Memory—Low Pair Associate	High risk	Low Risk	High Risk	Not detected	Not assessed	0 0 + + (Low)
	CVLT—Short Cued Recall	High risk	Low Risk	High Risk	Not detected	Not assessed	0 0 + + (Low)
	CVLT—Delayed Cued Recall	High risk	Low Risk	Low Risk	Not detected	Not assessed	0 + + + (moderate)
	CVLT—Recognition Memory	High risk	Low Risk	Low Risk	Not detected	Not assessed	0 + + + (moderate)
Verbal Intelligence	WAIS—Vocabulary	High risk	Low Risk	Low Risk	Not detected	Not assessed	0 + + + (moderate)
Performance intelligence	WAIS Nonverbal IQ	High risk	Low Risk	Low Risk	Not detected	Not assessed	0 + + + (moderate)

**Table 6.** Tests that are reported by single study and were not poolable.

Neurocognitive function	Cognitive test/subcomponent of cognitive test	PTSD+ vs. PTSD− survivor of wars		PTSD+ vs. Health Control		PTSD− vs. Health Control	
		Sample size	MD (95% CI)	Sample size	MD (95% CI)	Sample size	MD (95% CI)
Information processing speed	symbol Digit modalities (Hart 2008)	18	−1.77 [−2.92, −0.62]				
	WAIS—Digit Symbol (Green 2016)	18	−0.44 [−0.88, −0.01]				
visuo-spatial working memory	Corsi Block Tapping Test—Forward (Jelnik 2013)	44	−0.13 [−0.72, 0.47]	31	0.36 [−0.38, 1.10]	35	0.45 [−0.27, 1.17]
	Corsi Block Tapping Test—Backward (Jelnik 2013)	44	−0.48 [−1.08, 0.12]	31	−0.35 [−1.10, 0.39]	35	0.13 [−0.58, 0.85]
Language	COWAT (Phonemic ﬂuency) (Hart 2008)	18	−0.98 [−2.00, 0.03]				
	Boston Naming Test (Hart 2008)	18	−0.84 [−1.84, 0.16]				
	Groningen Intelligence Test (GIT) fluency (Wessel 2002)			40	−0.63 [−1.29, 0.02]		
	Animal Fluency—Semantic fluency (Hart 2008)	18	−0.39 [−1.35, 0.57]				
Learning and memory	Autobiographical positive cues (Wessel 2002)			40	−1.52 [−2.25, −0.79]		
	Autobiographical negative cues (Wessel 2002)			40	−1.43 [−2.15, −0.71]		
	WMS—Logical memory—Thematic scores (Freeman 2006)	20	−0.08 [−0.96, 0.80]	16	−0.05 [−1.06, 0.96]	16	0.02 [−0.99, 1.04]
	WMS—Logical Memory—Recall score (Freeman 2006)	20	−0.56 [−1.46, 0.33]	16	−0.4 [−1.45, 0.61]	16	0.14 [−0.88, 1.15]

WAIS (Wechsler Adult Intelligence Scale); COWA (Controlled Oral Word Association Test); WMS (Wechsler Memory Scale)

Due to inconsistently reporting across the studies PTSD+ vs. healthy control was only noted in learning and memory functions. Most of the results remained consistent in comparing PTSD+ survivors of war with healthy controls. Performance on many neuropsychological tests that were initially nonsignificant between PTSD+ and PTSD− elderly survivors of war became significant when comparing PTSD+ elderly survivors of war with the control population not suffering from PTSD. Some variables such as explicit memory, short cued recall, delayed cued recall and delayed free recall were significant in PTSD+ survivors of war as compared to the healthy control that was non-significant in the PTSD+ survivor of wars vs. PTSD− survivors of war. This trend possibly indicates an association of sub-threshold neurocognitive deficits in PTSD− survivors of war; therefore, the performance on tests that are nonsignificant for PTSD+ vs. PTSD− survivors of war became apparent in the second comparison. This can be further supported by comparing the effect sizes for performance for explicit memory, RAVLT, and CVLT. PTSD+ survivors exhibited larger effect measures when compared to healthy controls than compared with PTSD− survivors of war. Many commonly used tests in practice such as COWA, fluency tests were not pooled due to not meeting our criteria as reported in 2 or more studies; however, as these tests are commonly used we reported them as a single study effect. Performance on tests measuring visuospatial function and language were not significant.

Our review also had a few limitations. One of the limitations of our review was that some studies Golier 2002 [43], Yehuda 2005 [46], Freeman [48], Jelinek [57], and Hart [34] were duplicates and analyzed data on the same population. To avoid overestimation in our pooled analysis, we restricted our analysis to tests that were not reported in the original studies. Secondly, most studies in our pooled analysis had a small sample size and did not account for premorbid IQ; therefore, results of analysis need to be interpreted cautiously as we were unable to explore for publication bias, moderator effect of premorbid IQ and subgroup analysis respectively. Estimation of premorbid intelligence is important to determine whether the change in the neurocognitive function is greater than one can expect or is due to the measurement errors [59].

As compared to previous reviews our review also had much strength. Firstly, Schuitevoerder et al. reported their results based on the main neurocognitive function. Different neuropsychological tests measuring the same neurocognitive function require mental process in detecting the specific neurocognitive function [60]. There is always a potential that one aspect of the cognition might be working adequately than the other, therefore, pooling based on the neuropsychological tests provided better interpretation. This pattern was also noticeable in our analysis. For example, TMT-A and WAIS digit span forward both measured attentions. But performance on TMT-A is more complex and further requires information processing speed, as compared to the digit span forward test. Similarly, when compared digit span forward vs. digit span backward, the digit span forward measures attention efficiency whereas digit span backward is the measure of executive function and dependent on the working memory and mental flexibility.

Both Qureshi et al. [11] and Schuitevoerder et al. [12] categorized their results according to the trauma type. We did not have restrictions based on the war trauma type, which potentially increases the generalizability of our results. Schuitevoerder et al. [12] also identified duplicate publications during their review and used the average effect estimate, which in our opinion, is potential for overestimation. We excluded the duplicate study with a small sample size from our analysis. We included studies that recruited outpatient PTSD survivors of wars as compared to previous reviews [11],[12], which included studies with inpatients patients with major systematic comorbidities such as coronary artery bypass graft. Our rationale to exclude studies with inpatient PTSD patients and other major systematic ailments was that these patients potentially had a complicated course of illness, which can be a potential confounder to describe the association of cognitive impairments in PTSD survivors of war. We explored the quality of evidence with GRADE across the pooled analysis to determine confidence in our outcomes. We performed three different analyses to explore the possible effect of age on the association of cognitive deficits in elderly PTSD+ survivors of war and the normal ageing process. As compared to Qureshi et al, we excluded patient suffering from PTSD due to other reasons such as MVA, surgical process, and injuries due to which we had a more homogenous population in our pooled analysis.

### 7.1. Implications and future directions

As compared to the general population, PTSD is more prevalent in the survivors of war [6],[9],[61]. The combat traumas in the elderly may persist long after the initial exposure and can invariably affect the neurocognitive functions requiring complex tasks, retrieval of information and memory. Understanding the neurocognitive impairment in elderly patients is vital because the age-related changes in cognitive function can further compound the neurocognitive impairment and activities of daily livings [62],[63]. Based on our findings, these deficits were more pronounced in function requiring information processing speed and executive function, inhibition, mental flexibility, and delayed retrieval of information. A potential explanation for deficits in information processing speed, executive function and memory is preoccupation with the traumatic thoughts, intrusion, flash back, nightmares and sleep disruption and avoidance in PTSD. As the emotional symptoms persist beyond many years and act viciously to allocate the information processing towards the fear and traumatic events [64]. This preponderance of information processing speed towards fear and traumatic thoughts potentially makes disengaging from the traumatic memory harder, slows the formation of new memory, planning and executive functions [64].

One of the key implications of our review is that merely providing the symptomatic treatment to elderly survivors of war suffering from PTSD may not suffice and requires detailed neurocognitive assessment. The neurocognitive impairment in elderly survivors of war can hasten the recovery process [64]–[66]. Understanding the extent of neurocognitive deficits can potentially facilitate to stratify the support and management plan for the elderly survivors of war. For example, various components of cognitive behavioral therapy involve recalling past events or describing traumatic scenes, which may trigger traumatic flash backs, sleep impairment and avoidance behavior. It is possible that intrusive thoughts or negative processing associated with the trauma may hasten the recovery process [67]. On the other hand, other treatment strategies such as support therapy, recreational therapy, or educational intervention require more intact mental processing, and cognitive ability to learn new information. Not addressing the issues with complex mental processing, mental flexibility, attention processes can hinder the effectiveness of psychotherapies. As patients with PTSD experiences emotional symptoms such as avoidance, intrusion, and flash back of traumatic memories.

In conclusion, elderly survivors of war with PTSD exhibited poor performance on a neuropsychological test that required complex functioning or delayed information retrieval. The relatively poor performance was noted on PTSD+ survivors of war in comparison to the healthy control group and PTSD− survivors of war. There is a need for good quality, studies with large sample sizes and controlling for important variables such as ages, and premorbid IQ. Future studies may consider performing multiple comparisons such as PTSD patients with comorbidities, other psychological conditions for a better understanding of neurocognitive deficits in PTSD patients particularly in elderly survivors of war.

## References

[1] Samuelson KW, Neylan TC, Metzler TJ (2006). Neuropsychological functioning in posttraumatic stress disorder and alcohol abuse. Neuropsychology.

[2] Vasterling JJ, Proctor SP, Amoroso P (2006). Neuropsychological Outcomes of Army Personnel Following Deployment to the Iraq War. JAMA.

[3] Schinka JA, Loewenstein DA, Raj A (2010). Defining mild cognitive impairment: impact of varying decision criteria on neuropsychological diagnostic frequencies and correlates. Am J Geriatr Psychiatry.

[4] Avorn J (1995). Medication use and the elderly: current status and opportunities. Health Aff (Millwood).

[5] Shenoy P, Harugeri A (2015). Elderly patients' participation in clinical trials. Perspect Clin Res.

[6] Hoge CW, Castro CA, Messer SC (2004). Combat duty in Iraq and Afghanistan, mental health problems, and barriers to care. N Engl J Med.

[7] Yaffe K, Vittinghoff E, Lindquist K (2010). Posttraumatic stress disorder and risk of dementia among US veterans. Arch Gen Psychiatry.

[8] Brandes D, Ben-Schachar G, Gilboa A (2002). PTSD symptoms and cognitive performance in recent trauma survivors. Psychiatry Res.

[9] Dohrenwend BP, Turner JB, Turse NA (2006). The psychological risks of Vietnam for U.S. veterans: a revisit with new data and methods. Science.

[10] Spiro A, Schnurr PP, Aldwin CM (1994). Combat-related posttraumatic stress disorder symptoms in older men. Psychol Aging.

[11] Qureshi SU, Long ME, Bradshaw MR (2011). Does PTSD Impair Cognition Beyond the Effect of Trauma?. J Neuropsychiatry Clin Neurosci.

[12] Schuitevoerder S, Rosen JW, Twamley EW (2013). A meta-analysis of cognitive functioning in older adults with PTSD. J Anxiety Disord.

[13] Scott Mackin R, Lesselyong JA, Yaffe K (2012). Pattern of cognitive impairment in older veterans with posttraumatic stress disorder evaluated at a memory disorders clinic. Int J Geriatr Psychiatry.

[14] Sapolsky RM (2000). Glucocorticoids and hippocampal atrophy in neuropsychiatric disorders. Arch Gen Psychiatry.

[15] Van Achterberg ME, Rohrbaugh RM, Southwick SM (2001). Emergence of PTSD in trauma survivors with dementia. J Clin Psychiatry.

[16] Mittal D, Torres R, Abashidze A (2001). Worsening of post-traumatic stress disorder symptoms with cognitive decline: case series. J Geriatr Psychiatry Neurol.

[17] Johnston D (2000). A series of cases of dementia presenting with PTSD symptoms in World War II combat veterans. J Am Geriatr Soc.

[18] Cook JM, Ruzek JI, Cassidy E (2003). Practical Geriatrics: Possible Association of Posttraumatic Stress Disorder With Cognitive Impairment Among Older Adults. Psychiatr Serv.

[19] Boscarino JA (2004). Posttraumatic stress disorder and physical illness: results from clinical and epidemiologic studies. Ann N Y Acad Sci.

[20] Drescher KD, Rosen CS, Burling TA (2003). Causes of death among male veterans who received residential treatment for PTSD. J Trauma Stress.

[21] Zatzick DF, Marmar CR, Weiss DS (1997). Posttraumatic stress disorder and functioning and quality of life outcomes in a nationally representative sample of male Vietnam veterans. Am J Psychiatry.

[22] Chaplin R (2000). Psychiatrists can cause stigma too. Br J Psychiatry.

[23] Kleim B, Ehlers A (2008). Reduced autobiographical memory specificity predicts depression and posttraumatic stress disorder after recent trauma. J Consult Clin Psychol.

[24] Levine ME (2012). Modeling the Rate of Senescence: Can Estimated Biological Age Predict Mortality More Accurately Than Chronological Age?. J Gerontol A Biol Sci Med Sci.

[25] Harvey PD (2019). Domains of cognition and their assessment. Dialogues Clin Neurosci.

[26] Sachdev PS, Blacker D, Blazer DG (2014). Classifying neurocognitive disorders: the DSM-5 approach. Nat Rev Neurol.

[27] Moore SA, Zoellner LA (2007). Overgeneral autobiographical memory and traumatic events: an evaluative review. Psychol Bull.

[28] Guez J, Naveh-Benjamin M, Yankovsky Y (2011). Traumatic stress is linked to a deficit in associative episodic memory. J Trauma Stress.

[29] Lagarde G, Doyon J, Brunet A (2010). Memory and executive dysfunctions associated with acute posttraumatic stress disorder. Psychiatry Res.

[30] Kanagaratnam P, Asbjørnsen AE (2007). Executive deficits in chronic PTSD related to political violence. J Anxiety Disord.

[31] Sachinvala N, Von Scotti H, McGuire M (2000). Memory, attention, function, and mood among patients with chronic posttraumatic stress disorder. J Nerv Ment Dis.

[32] Vasterling JJ, Duke LM, Brailey K (2002). Attention, learning, and memory performances and intellectual resources in Vietnam veterans: PTSD and no disorder comparisons. Neuropsychology.

[33] Green E, Fairchild JK, Kinoshita LM (2015). Effects of Posttraumatic Stress Disorder and Metabolic Syndrome on Cognitive Aging in Veterans. Gerontologist.

[34] Hart J, Kimbrell T, Fauver P (2008). Cognitive Dysfunctions Associated With PTSD: Evidence from World War II Prisoners of War. J Neuropsychiatry Clin Neurosci.

[35] Yehuda R, Golier JA, Tischler L (2007). Hippocampal volume in aging combat veterans with and without post-traumatic stress disorder: relation to risk and resilience factors. J Psychiatr Res.

[36] Liberati A, Altman DG, Tetzlaff J (2009). The PRISMA statement for reporting systematic reviews and meta-analyses of studies that evaluate healthcare interventions: explanation and elaboration. BMJ.

[37] Singh S, Bajorek B (2014). Defining ‘elderly’ in clinical practice guidelines for pharmacotherapy. Pharm Pract (Granada).

[38] Harugeri A, Joseph J, Parthasarathi G (2010). Potentially inappropriate medication use in elderly patients: a study of prevalence and predictors in two teaching hospitals. J Postgrad Med.

[39] Weathers FW, Bovin MJ, Lee DJ (2018). The Clinician-Administered PTSD Scale for DSM-5 (CAPS-5): Development and initial psychometric evaluation in military veterans. Psychol Assess.

[40] McCarthy S (2008). Post-Traumatic Stress Diagnostic Scale (PDS). Occup Med (Lond).

[41] Foa EB, McLean CP, Zang Y (2016). Psychometric properties of the Posttraumatic Stress Disorder Symptom Scale Interview for DSM-5 (PSSI-5). Psychol Assess.

[42] Ruglass LM, Papini S, Trub L (2014). Psychometric Properties of the Modified Posttraumatic Stress Disorder Symptom Scale among Women with Posttraumatic Stress Disorder and Substance Use Disorders Receiving Outpatient Group Treatments. J Trauma Stress Disord Treat.

[43] Golier JA, Yehuda R, Lupien SJ (2002). Memory performance in Holocaust survivors with posttraumatic stress disorder. Am J Psychiatry.

[44] Golier JA, Yehuda R, Lupien SJ (2003). Memory for trauma-related information in Holocaust survivors with PTSD. Psychiatry Res.

[45] Golier JA, Yehuda R, De Santi S (2005). Absence of hippocampal volume differences in survivors of the Nazi Holocaust with and without posttraumatic stress disorder. Psychiatry Res.

[46] Yehuda R, Golier JA, Harvey PD (2005). Relationship between cortisol and age-related memory impairments in Holocaust survivors with PTSD. Psychoneuroendocrinology.

[47] Yehuda R, Golier JA, Halligan SL (2004). Learning and memory in Holocaust survivors with posttraumatic stress disorder. Biol Psychiatry.

[48] Freeman T, Kimbrell T, Booe L (2006). Evidence of resilience: Neuroimaging in former prisoners of war. Psychiatry Res.

[49] Yehuda R, Tischler L, Golier JA (2006). Longitudinal assessment of cognitive performance in Holocaust survivors with and without PTSD. Biol Psychiatry.

[50] Agency for Healthcare Research and Quality (US), Rockville (MD) (2014). Cognitive Outcomes After Cardiovascular Procedures in Older Adults: A Systematic Review.

[51] Parkinson WL, Rehman Y, Rathbone M (2020). Performances on individual neurocognitive tests by people experiencing a current major depression episode: A systematic review and meta-analysis. J Affective Disord.

[52] Rathbone M, Parkinson W, Rehman Y (2016). Magnitude and variability of effect sizes for the associations between chronic pain and cognitive test performances: a meta-analysis. Br J Pain.

[53] Wells G, Shea B, O'connell D (2014). The Newcastle-Ottawa Scale (NOS) for Assessing the Quality of Nonrandomised Studies in Meta-Analyses.

[54] Guyatt GH, Oxman AD, Vist GE (2008). GRADE: an emerging consensus on rating quality of evidence and strength of recommendations. BMJ.

[55] Wittekind CE, Jelinek L, Kellner M (2010). Intergenerational transmission of biased information processing in posttraumatic stress disorder (PTSD) following displacement after World War II. J Anxiety Disord.

[56] Golier JA, Harvey PD, Legge J (2006). Memory performance in older trauma survivors: implications for the longitudinal course of PTSD. Ann N Y Acad Sci.

[57] Jelinek L, Wittekind CE, Moritz S (2013). Neuropsychological functioning in posttraumatic stress disorder following forced displacement in older adults and their offspring. Psychiatry Res.

[58] Wessel I, Merckelbach H, Dekkers T (2002). Autobiographical Memory Specificity, Intrusive Memory, and General Memory Skills in Dutch–Indonesian Survivors of the World War II Era. J Trauma Stress.

[59] Schoenberg MR, Lange RT, Marsh P, Kreutzer JS, DeLuca J, Caplan B (2011). Premorbid Intelligence. Encyclopedia of Clinical Neuropsychology.

[60] Cicerone KD, Azulay J (2002). Diagnostic utility of attention measures in postconcussion syndrome. Clin Neuropsychol.

[61] Munjiza J, Britvic D, Radman M (2017). Severe war-related trauma and personality pathology: a case-control study. BMC Psychiatry.

[62] Murman DL (2015). The Impact of Age on Cognition. Semin Hear.

[63] Lapp LK, Agbokou C, Ferreri F (2011). PTSD in the elderly: the interaction between trauma and aging. Int Psychogeriatr.

[64] Hayes JP, Vanelzakker MB, Shin LM (2012). Emotion and cognition interactions in PTSD: a review of neurocognitive and neuroimaging studies. Front Integr Neurosci.

[65] Ben-Zion Z, Fine NB, Keynan NJ (2018). Cognitive Flexibility Predicts PTSD Symptoms: Observational and Interventional Studies. Front Psychiatry.

[66] Tanev KS, Federico LE, Terry DP (2019). Cognitive Impairment and Predicting Response to Treatment in an Intensive Clinical Program for Post-9/11 Veterans With Posttraumatic Stress Disorder. J Neuropsychiatry Clin Neurosci.

[67] Kessler RC, Berglund P, Demler O (2005). Lifetime Prevalence and Age-of-Onset Distributions of DSM-IV Disorders in the National Comorbidity Survey Replication. Arch Gen Psychiatry.

