# Supplementary material for: The extent of the neurocognitive impairment in elderly survivors of war suffering from PTSD: meta-analysis and literature review
Source: AIMS Neurosci. 2020 Nov 25;8(1):47–73. doi: 10.3934/Neuroscience.2021003 (PMC7815483; doi:10.3934/Neuroscience.2021003)
Supplement: Supplementary file 1 [file neurosci-08-01-003-s001.pdf]

AIMS Neuroscience

AIMS Neuroscience, 8(1): 47–73.
DOI: 10.3934/Neuroscience.2021003
Received: 27 August 2020
Accepted: 23 November 2020
Published: 25 November 2020

http://www.aimspress.com/journal/aimsph

Review

The extent of the neurocognitive impairment in elderly survivors of war suffering from PTSD: meta-analysis and literature review

Yasir Rehman[1,*], Cindy Zhang[2], Haolin Ye[3], Lionel Fernandes[3], Mathieu Marek[3], Andrada Cretu[3] and William Parkinson[4]

[1] Health Research Methodology, McMaster University, Hamilton, ON, Canada
[2] Faculty of Health Sciences, McMaster University, Hamilton ON, Canada
[3] Faculty of Life Sciences, McMaster University, Hamilton ON, Canada
[4] School of Rehabilitation Science, McMaster University, Hamilton ON, Canada

* **Correspondence:** Email: dry_rehman@yahoo.ca; Tel: 2893397848.

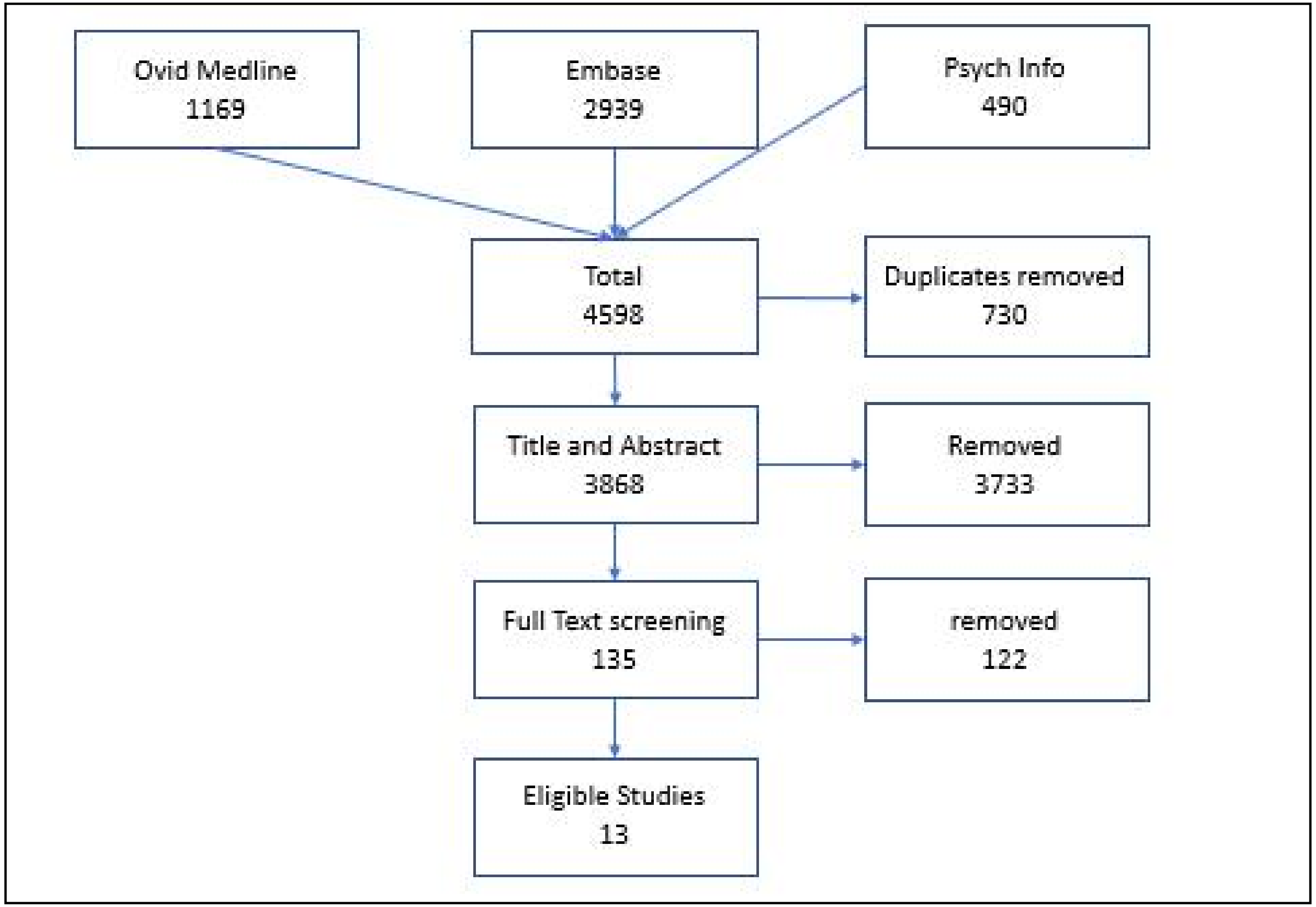

Figure 1: PRISMA Flow Chart showing the number of articles collected for the screening process

Attention and Information processing speed

Figure 2 (a): TMT-A (PTSD+ vs. PTSD- survivors of war)

Study or Subgroup	PTSD- survivors of war Mean	SD	Total	PTSD+ survivors of war Mean	SD	Total	Weight	Mean Difference IV, Random, 95% CI
Green 2016	29.3	7.3	33	37.5	11.7	55	62.1%	-8.20 [-12.17, -4.23]
Hart 2008	48	18.1	11	56.3	16.3	7	8.7%	-8.30 [-24.43, 7.83]
Jelinek 2013	44.46	11.93	24	45.25	14.12	20	29.1%	-0.79 [-8.61, 7.03]
**Total (95% CI)**			**68**			**82**	**100.0%**	**-6.05 [-11.04, -1.06]**

Heterogeneity: Tau² = 6.33; Chi² = 2.78, df = 2 (P = 0.25); I² = 28%
Test for overall effect: Z = 2.38 (P = 0.02)

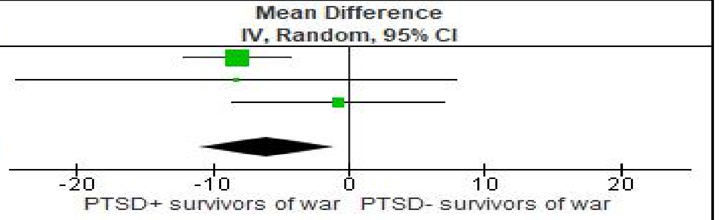

Figure 2 (b): Digit Span Forward (PTSD+ vs. PTSD- survivors of war)

Study or Subgroup	PTSD- survivors of war Mean	SD	Total	PTSD+ survivors of war Mean	SD	Total	Weight	Mean Difference IV, Fixed, 95% CI
Hart 2008	11.2	1.9	11	11	5.2	7	8.4%	0.20 [-3.81, 4.21]
Jelinek 2013	7	2.09	24	7.1	2.02	20	91.6%	-0.10 [-1.32, 1.12]
**Total (95% CI)**			**35**			**27**	**100.0%**	**-0.07 [-1.24, 1.09]**

Heterogeneity: Chi² = 0.02, df = 1 (P = 0.89); I² = 0%
Test for overall effect: Z = 0.13 (P = 0.90)

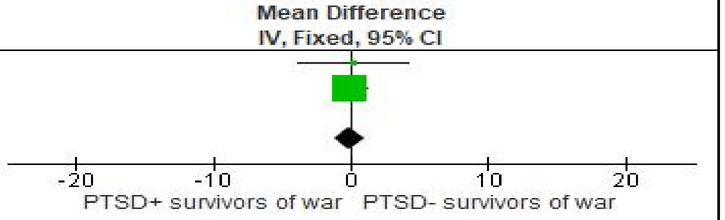

Executive function

Figure 3 (a): TMT-B (PTSD+ vs. PTSD- survivors of war)

Study or Subgroup	PTSD- survivors of war Mean	SD	Total	PTSD+ survivors of war Mean	SD	Total	Weight	Mean Difference IV, Random, 95% CI
Green 2016	68.5	24.1	33	88.8	52.7	55	45.4%	-20.30 [-36.47, -4.13]
Hart 2008	94.3	30.3	11	147.2	36.7	7	21.3%	-52.90 [-85.45, -20.35]
Jelinek 2013	97.27	41.17	24	113.23	35.71	20	33.3%	-15.96 [-38.68, 6.76]
**Total (95% CI)**			**68**			**82**	**100.0%**	**-25.80 [-43.70, -7.89]**

Heterogeneity: Tau² = 115.93; Chi² = 3.71, df = 2 (P = 0.16); I² = 46%
Test for overall effect: Z = 2.82 (P = 0.005)

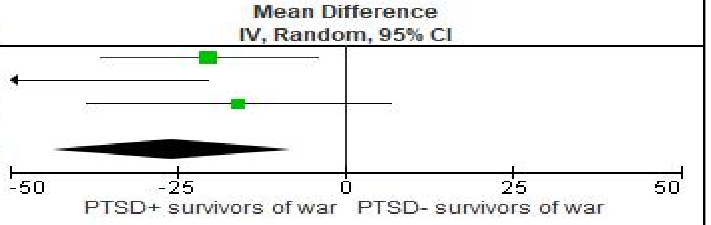

Figure 3 (b): Digit span backward (PTSD+ vs. PTSD- survivors of war)

Study or Subgroup	PTSD+ survivors of war Mean	SD	Total	PTSD- survivors of war Mean	SD	Total	Weight	Mean Difference IV, Fixed, 95% CI
Hart 2008	5.1	1.8	7	6.6	1.6	11	24.4%	-1.50 [-3.13, 0.13]
Jelinek 2013	5.4	1.54	20	6	1.59	24	75.6%	-0.60 [-1.53, 0.33]
**Total (95% CI)**			**27**			**35**	**100.0%**	**-0.82 [-1.63, -0.01]**

Heterogeneity: Chi² = 0.88, df = 1 (P = 0.35); I² = 0%
Test for overall effect: Z = 1.99 (P = 0.05)

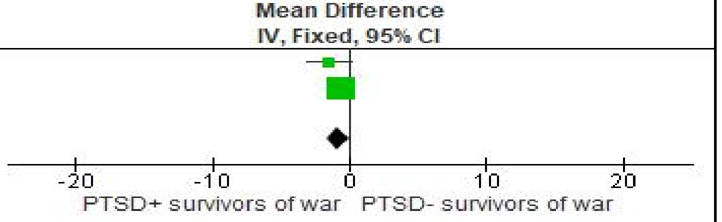

Figure 3 (c): Stroop color word test (PTSD+ vs. PTSD- survivors of war)

Study or Subgroup	PTSD- survivors of war Mean	SD	Total	PTSD+ survivors of war Mean	SD	Total	Weight	Mean Difference IV, Fixed, 95% CI
Green 2016	36.2	11.8	33	44.5	27	55	99.0%	-8.30 [-16.49, -0.11]
Wittekind 2010	287.48	164.85	24	300.38	110.39	22	1.0%	-12.90 [-93.38, 67.58]
**Total (95% CI)**			**57**			**77**	**100.0%**	**-8.35 [-16.50, -0.20]**

Heterogeneity: Chi² = 0.01, df = 1 (P = 0.91); I² = 0%
Test for overall effect: Z = 2.01 (P = 0.04)

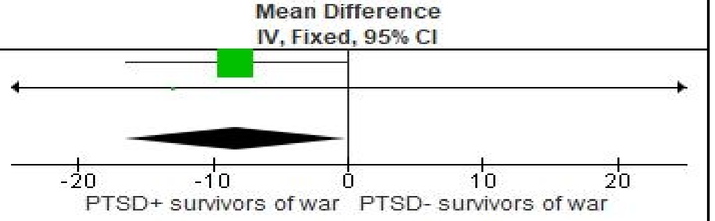

Learning and memory

Figure 4 (a): Explicit memory high pair associate- PTSD+ vs. PTSD-

	PTSD+ survivors of war			PTSD- survivors of war				Mean Difference	Mean Difference
Study or Subgroup	Mean	SD	Total	Mean	SD	Total	Weight	IV, Fixed, 95% CI	IV, Fixed, 95% CI
Golier 2002	9.08	2.08	31	10.19	1.65	16	34.5%	-1.11 [-2.20, -0.02]	
Yehuda 2006	3.71	1.1	14	4.06	1	13	65.5%	-0.35 [-1.14, 0.44]	
**Total (95% CI)**			**45**			**29**	**100.0%**	**-0.61 [-1.25, 0.03]**	
Heterogeneity: Chi² = 1.22, df = 1 (P = 0.27); I² = 18%									
Test for overall effect: Z = 1.87 (P = 0.06)									

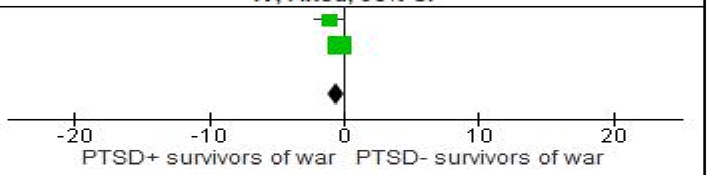

Figure 4 (b): Explicit memory low pair associate- PTSD+ vs. PTSD-

	PTSD+ survivors of war			PTSD- survivors of war				Mean Difference	Mean Difference
Study or Subgroup	Mean	SD	Total	Mean	SD	Total	Weight	IV, Fixed, 95% CI	IV, Fixed, 95% CI
Golier 2002	3.76	2.89	31	6.84	3.16	16	29.8%	-3.08 [-4.93, -1.23]	
Yehuda 2006	2.25	1.7	14	3.28	1.5	13	70.2%	-1.03 [-2.24, 0.18]	
**Total (95% CI)**			**45**			**29**	**100.0%**	**-1.64 [-2.65, -0.63]**	
Heterogeneity: Chi² = 3.30, df = 1 (P = 0.07); I² = 70%									
Test for overall effect: Z = 3.18 (P = 0.001)									

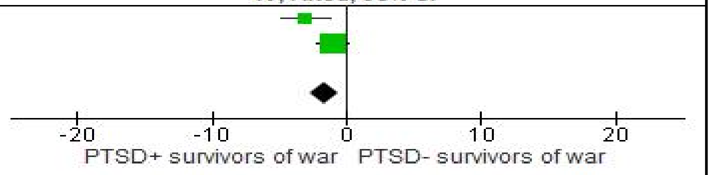

Figure 4 (c): Explicit memory high pair associate- PTSD+ vs. Control

	PTSD+ survivors of war			Healthy Control				Mean Difference	Mean Difference
Study or Subgroup	Mean	SD	Total	Mean	SD	Total	Weight	IV, Fixed, 95% CI	IV, Fixed, 95% CI
Golier 2002	9.08	2.08	31	10.83	1.24	35	41.3%	-1.75 [-2.59, -0.91]	
Yehuda 2006	3.71	1.1	14	5	0.9	19	58.7%	-1.29 [-1.99, -0.59]	
**Total (95% CI)**			**45**			**54**	**100.0%**	**-1.48 [-2.02, -0.94]**	
Heterogeneity: Chi² = 0.68, df = 1 (P = 0.41); I² = 0%									
Test for overall effect: Z = 5.38 (P < 0.00001)									

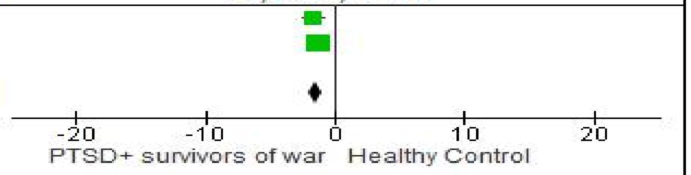

Figure 4 (d): Explicit memory low pair associate- PTSD+ vs. Control

	PTSD+ survivors of war			Healthy Control				Mean Difference	Mean Difference
Study or Subgroup	Mean	SD	Total	Mean	SD	Total	Weight	IV, Fixed, 95% CI	IV, Fixed, 95% CI
Golier 2002	3.76	2.89	31	7	3.12	35	38.4%	-3.24 [-4.69, -1.79]	
Yehuda 2006	2.25	1.7	14	2.84	1.6	19	61.6%	-0.59 [-1.73, 0.55]	
**Total (95% CI)**			**45**			**54**	**100.0%**	**-1.61 [-2.51, -0.71]**	
Heterogeneity: Chi² = 7.90, df = 1 (P = 0.005); I² = 87%									
Test for overall effect: Z = 3.51 (P = 0.0005)									

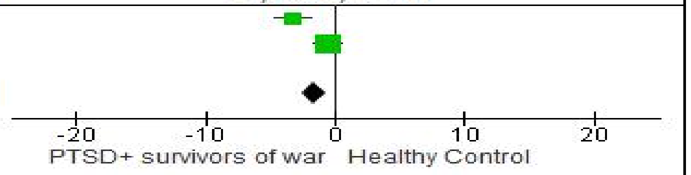

Figure 4 (e): Explicit memory high pair associate- PTSD- vs. Control

	PTSD- survivors of war			Healthy Control				Mean Difference	Mean Difference
Study or Subgroup	Mean	SD	Total	Mean	SD	Total	Weight	IV, Fixed, 95% CI	IV, Fixed, 95% CI
Golier 2002	10.19	1.65	16	10.83	1.24	35	86.7%	-0.64 [-1.55, 0.27]	
Yehuda 2006	4.06	1	13	2.84	5	19	13.3%	1.22 [-1.09, 3.53]	
**Total (95% CI)**			**29**			**54**	**100.0%**	**-0.39 [-1.24, 0.45]**	
Heterogeneity: Chi² = 2.15, df = 1 (P = 0.14); I² = 54%									
Test for overall effect: Z = 0.91 (P = 0.36)									

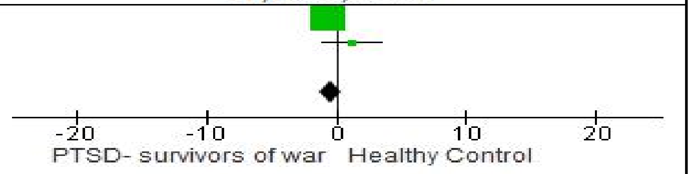

Figure 4 (f): Explicit memory Low pair associate- PTSD- vs. Control

	Healthy Control			PTSD- survivors of war				Mean Difference	Mean Difference
Study or Subgroup	Mean	SD	Total	Mean	SD	Total	Weight	IV, Fixed, 95% CI	IV, Fixed, 95% CI
Golier 2002	7	3.12	35	6.84	3.16	16	25.4%	0.16 [-1.70, 2.02]	
Yehuda 2006	2.84	1.6	19	3.28	1.5	13	74.6%	-0.44 [-1.53, 0.65]	
**Total (95% CI)**			**54**			**29**	**100.0%**	**-0.29 [-1.23, 0.65]**	
Heterogeneity: Chi² = 0.30, df = 1 (P = 0.59); I² = 0%									
Test for overall effect: Z = 0.60 (P = 0.55)									

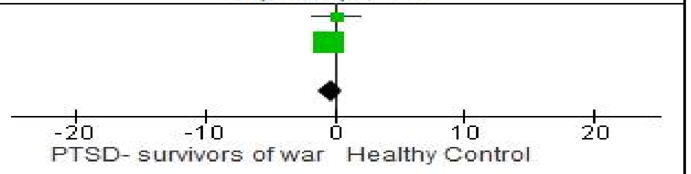

Learning and memory

Figure 5 (a): Implicit memory high pair associate- PTSD+ vs. PTSD-

Study or Subgroup	PTSD- survivors of war Mean	SD	Total	PTSD+ survivors of war Mean	SD	Total	Weight	Mean Difference IV, Fixed, 95% CI
Yehuda 2005	43.2	15	16	50	12.5	19	2.2%	-6.80 [-16.05, 2.45]
Yehuda 2006	5.08	2.2	13	5.33	1.4	14	97.8%	-0.25 [-1.65, 1.15]
**Total (95% CI)**			**29**			**33**	**100.0%**	**-0.40 [-1.78, 0.99]**

Heterogeneity: Chi² = 1.88, df = 1 (P = 0.17); I² = 47%
Test for overall effect: Z = 0.56 (P = 0.57)

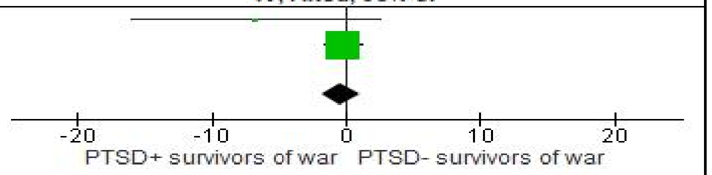

Figure 5 (b): Implicit memory low pair associate- PTSD+ vs. PTSD-

Study or Subgroup	PTSD- survivors of war Mean	SD	Total	PTSD+ survivors of war Mean	SD	Total	Weight	Mean Difference IV, Fixed, 95% CI
Yehuda 2005	42.2	12.3	16	48	16.8	19	3.0%	-5.80 [-15.46, 3.86]
Yehuda 2006	5	2.4	13	5.75	2.1	14	97.0%	-0.75 [-2.46, 0.96]
**Total (95% CI)**			**29**			**33**	**100.0%**	**-0.90 [-2.58, 0.78]**

Heterogeneity: Chi² = 1.02, df = 1 (P = 0.31); I² = 2%
Test for overall effect: Z = 1.05 (P = 0.29)

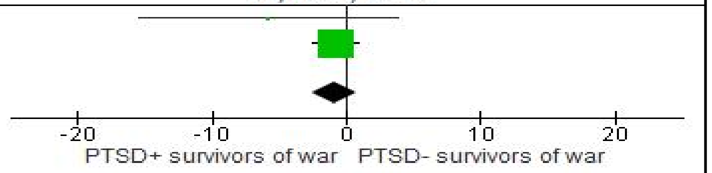

Figure 5 (c): Implicit memory high pair associate- PTSD+ vs. Control

Study or Subgroup	PTSD+ survivors of war Mean	SD	Total	Healthy Control Mean	SD	Total	Weight	Mean Difference IV, Fixed, 95% CI
Yehuda 2005	48.2	20.5	28	50	12.5	19	1.9%	-1.80 [-11.25, 7.65]
Yehuda 2006	5.31	2.4	19	5.33	1.4	14	98.1%	-0.02 [-1.32, 1.28]
**Total (95% CI)**			**47**			**33**	**100.0%**	**-0.05 [-1.35, 1.24]**

Heterogeneity: Chi² = 0.13, df = 1 (P = 0.71); I² = 0%
Test for overall effect: Z = 0.08 (P = 0.94)

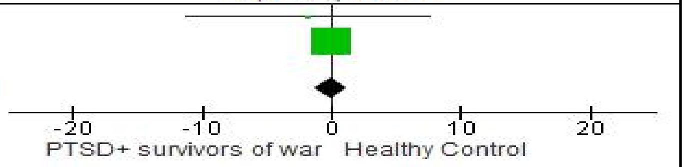

Figure 5 (d): Implicit memory low pair associate- PTSD+ vs. Control

Study or Subgroup	PTSD+ survivors of war Mean	SD	Total	Healthy Control Mean	SD	Total	Weight	Mean Difference IV, Fixed, 95% CI
Yehuda 2005	44.6	19.4	28	48	16.8	19	2.2%	-3.40 [-13.83, 7.03]
Yehuda 2006	4.95	2.5	19	5.75	2.1	14	97.8%	-0.80 [-2.37, 0.77]
**Total (95% CI)**			**47**			**33**	**100.0%**	**-0.86 [-2.41, 0.70]**

Heterogeneity: Chi² = 0.23, df = 1 (P = 0.63); I² = 0%
Test for overall effect: Z = 1.08 (P = 0.28)

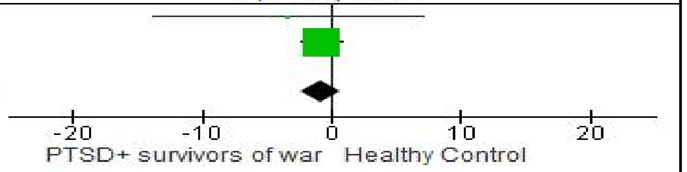

Figure 5 (e): Implicit memory high pair associate- PTSD- vs. Control

Study or Subgroup	PTSD- survivors of war Mean	SD	Total	Healthy Control Mean	SD	Total	Weight	Mean Difference IV, Fixed, 95% CI
Yehuda 2005	43.2	1.5	16	48.2	20.5	28	4.3%	-5.00 [-12.63, 2.63]
Yehuda 2006	5.08	2.2	13	5.31	2.4	19	95.7%	-0.23 [-1.84, 1.38]
**Total (95% CI)**			**29**			**47**	**100.0%**	**-0.43 [-2.01, 1.14]**

Heterogeneity: Chi² = 1.44, df = 1 (P = 0.23); I² = 30%
Test for overall effect: Z = 0.54 (P = 0.59)

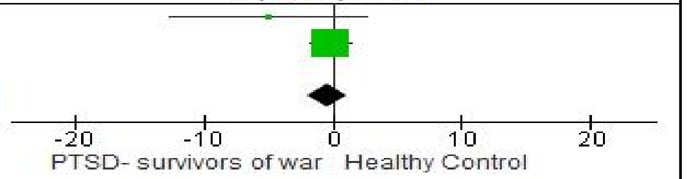

Figure 5 (f): Implicit memory Low pair associate- PTSD- vs. Control

Study or Subgroup	PTSD- survivors of war Mean	SD	Total	Healthy Control Mean	SD	Total	Weight	Mean Difference IV, Fixed, 95% CI
Yehuda 2005	42.2	12.3	16	44.6	19.4	28	3.3%	-2.40 [-11.78, 6.98]
Yehuda 2006	5	2.4	13	4.95	2.5	19	96.7%	0.05 [-1.67, 1.77]
**Total (95% CI)**			**29**			**47**	**100.0%**	**-0.03 [-1.72, 1.66]**

Heterogeneity: Chi² = 0.25, df = 1 (P = 0.61); I² = 0%
Test for overall effect: Z = 0.03 (P = 0.97)

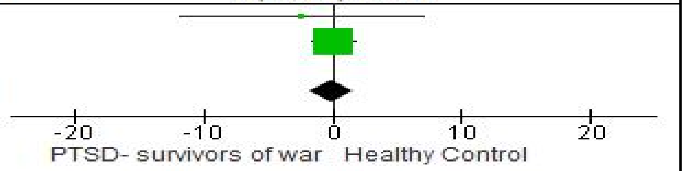

Learning and memory (verbal memory)

Figure 6 (a): RAVLT- Immediate Recall (PTSD+ vs. PTSD-)

Study or Subgroup	PTSD+ survivors of war Mean	SD	Total	PTSD- survivors of war Mean	SD	Total	Weight	Mean Difference IV, Fixed, 95% CI
Freeman 2016	8.7	2.2	10	8.6	2.5	10	37.7%	0.10 [-1.96, 2.16]
Yehuda 2004	7.9	3.3	36	9.2	3.1	26	62.3%	-1.30 [-2.91, 0.31]
**Total (95% CI)**			**46**			**36**	**100.0%**	**-0.77 [-2.04, 0.50]**
Heterogeneity: Chi² = 1.10, df = 1 (P = 0.29); I² = 9%								
Test for overall effect: Z = 1.19 (P = 0.23)								

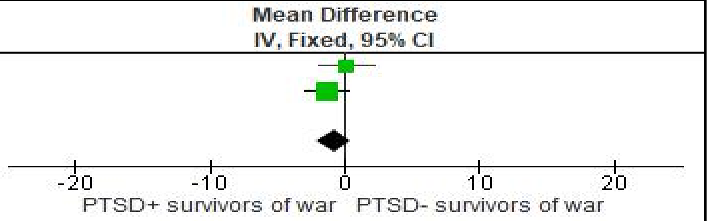

Figure 6 (b): RAVLT- Delayed Recall (PTSD+ vs. PTSD-)

Study or Subgroup	PTSD+ survivors of war Mean	SD	Total	PTSD- survivors of war Mean	SD	Total	Weight	Mean Difference IV, Fixed, 95% CI
Freeman 2016	33.3	6.9	10	34.1	10.1	10	1.2%	-0.80 [-8.38, 6.78]
Green 2016	7.4	2.9	55	8.1	1.9	33	66.7%	-0.70 [-1.70, 0.30]
Yehuda 2004	8.2	3.6	36	9.8	3.7	26	19.7%	-1.60 [-3.45, 0.25]
Yehuda 2006	8.5	3.6	14	10	2.5	13	12.4%	-1.50 [-3.82, 0.82]
**Total (95% CI)**			**115**			**82**	**100.0%**	**-0.98 [-1.80, -0.16]**
Heterogeneity: Chi² = 0.93, df = 3 (P = 0.82); I² = 0%								
Test for overall effect: Z = 2.34 (P = 0.02)								

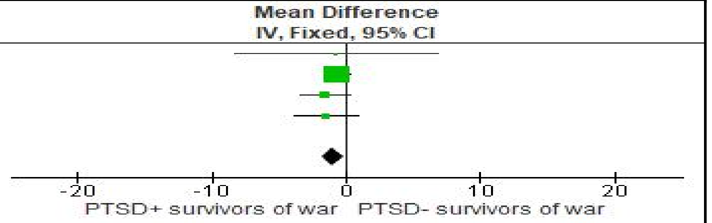

Figure 6 (c): RAVLT- Immediate Recall (PTSD+ vs. Healthy Control)

Study or Subgroup	PTSD+ survivors of war Mean	SD	Total	Healthy Control Mean	SD	Total	Weight	Mean Difference IV, Random, 95% CI
Freeman 2016	8.7	2.2	10	8.7	2.3	6	24.8%	0.00 [-2.29, 2.29]
Wessel 2002	4.9	1.8	25	5.3	1.7	15	39.7%	-0.40 [-1.51, 0.71]
Yehuda 2004	7.9	3.3	36	10.5	3	40	35.5%	-2.60 [-4.02, -1.18]
**Total (95% CI)**			**71**			**61**	**100.0%**	**-1.08 [-2.70, 0.54]**
Heterogeneity: Tau² = 1.40; Chi² = 6.67, df = 2 (P = 0.04); I² = 70%								
Test for overall effect: Z = 1.31 (P = 0.19)								

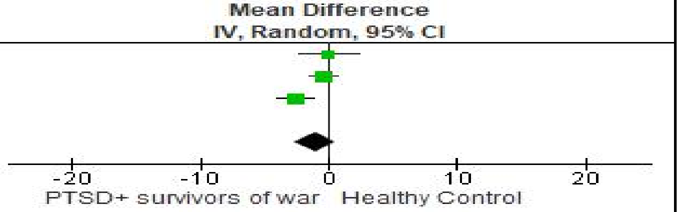

Figure 6 (d): RAVLT- Delayed Recall (PTSD+ vs. Healthy Control)

Study or Subgroup	PTSD+ survivors of war Mean	SD	Total	PTSD- survivors of war Mean	SD	Total	Weight	Mean Difference IV, Random, 95% CI
Freeman 2016	33.3	6.9	10	34.5	8.9	6	1.5%	-1.20 [-9.51, 7.11]
Wessel 2002	8.3	2.8	25	9.5	2.3	15	39.4%	-1.20 [-2.80, 0.40]
Yehuda 2004	8.2	3.6	36	10.5	3	40	44.9%	-2.30 [-3.80, -0.80]
Yehuda 2006	8.5	3.6	14	10	4.2	19	14.2%	-1.50 [-4.17, 1.17]
**Total (95% CI)**			**85**			**80**	**100.0%**	**-1.74 [-2.74, -0.73]**
Heterogeneity: Tau² = 0.00; Chi² = 1.02, df = 3 (P = 0.80); I² = 0%								
Test for overall effect: Z = 3.39 (P = 0.0007)								

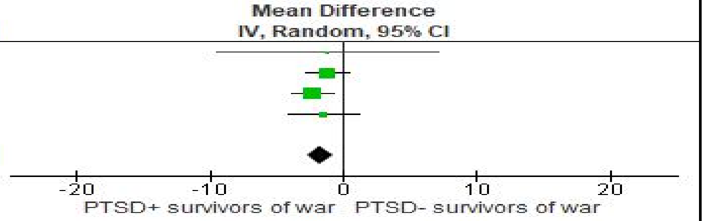

Figure 6 (e): RAVLT- Immediate Recall (PTSD- vs. Healthy Control)

Study or Subgroup	PTSD- survivors of war Mean	SD	Total	Healthy Control Mean	SD	Total	Weight	Mean Difference IV, Fixed, 95% CI
Freeman 2016	8.6	2.5	10	8.7	2.3	6	28.3%	-0.10 [-2.51, 2.31]
Yehuda 2004	9.2	3.1	26	10.5	3	40	71.7%	-1.30 [-2.81, 0.21]
**Total (95% CI)**			**36**			**46**	**100.0%**	**-0.96 [-2.24, 0.32]**
Heterogeneity: Chi² = 0.69, df = 1 (P = 0.41); I² = 0%								
Test for overall effect: Z = 1.47 (P = 0.14)								

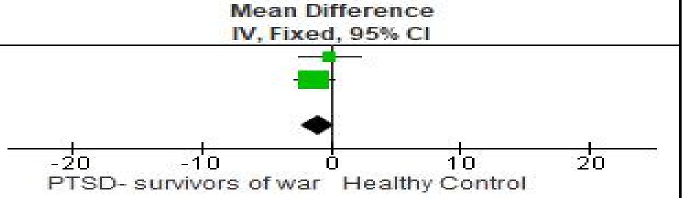

Figure 6 (f): RAVLT- Delayed Recall (PTSD- vs. Healthy Control)

Study or Subgroup	PTSD- survivors of war Mean	SD	Total	Healthy Control Mean	SD	Total	Weight	Mean Difference IV, Random, 95% CI
Freeman 2016	34.1	10.1	10	34.5	8.9	6	2.1%	-0.40 [-9.88, 9.08]
Yehuda 2004	9.8	3.7	26	10.5	3	40	63.9%	-0.70 [-2.40, 1.00]
Yehuda 2006	10	2.5	13	10	4.2	19	34.1%	0.00 [-2.33, 2.33]
**Total (95% CI)**			**49**			**65**	**100.0%**	**-0.46 [-1.81, 0.90]**
Heterogeneity: Tau² = 0.00; Chi² = 0.23, df = 2 (P = 0.89); I² = 0%								
Test for overall effect: Z = 0.66 (P = 0.51)								

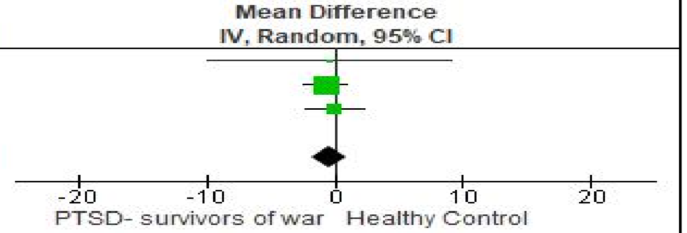

Learning and memory (verbal memory- cued recall)

Figure 7 (a): CVLT- Short Recall (PTSD+ vs. PTSD-

Study or Subgroup	PTSD+ survivors of war Mean	SD	Total	PTSD- survivors of war Mean	SD	Total	Weight	Mean Difference IV, Fixed, 95% CI
Yehuda 2004	9.57	3.2	14	10.23	2.2	13	41.0%	-0.66 [-2.72, 1.40]
Yehuda 2006	9.2	3.4	36	10.5	3.4	26	59.0%	-1.30 [-3.02, 0.42]
**Total (95% CI)**			**50**			**39**	**100.0%**	**-1.04 [-2.36, 0.28]**
Heterogeneity: Chi² = 0.22, df = 1 (P = 0.64); I² = 0%								
Test for overall effect: Z = 1.54 (P = 0.12)								

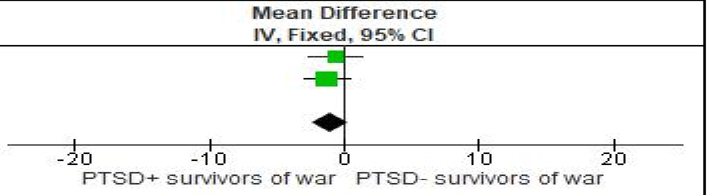

Figure 7 (b): CVLT- Delayed Recall (PTSD+ vs. PTSD-)

Study or Subgroup	PTSD+ survivors of war Mean	SD	Total	PTSD- survivors of war Mean	SD	Total	Weight	Mean Difference IV, Fixed, 95% CI
Yehuda 2004	12.9	2.9	36	14	1.8	26	80.1%	-1.10 [-2.27, 0.07]
Yehuda 2006	9.57	3.8	14	10.78	2.3	13	19.9%	-1.21 [-3.56, 1.14]
**Total (95% CI)**			**50**			**39**	**100.0%**	**-1.12 [-2.17, -0.07]**
Heterogeneity: Chi² = 0.01, df = 1 (P = 0.93); I² = 0%								
Test for overall effect: Z = 2.09 (P = 0.04)								

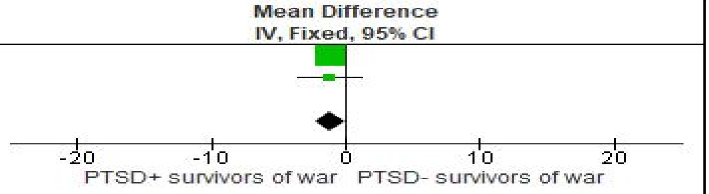

Figure 7 (c): CVLT- Short Recall (PTSD+ vs. Healthy Control)

Study or Subgroup	PTSD+ survivors of war Mean	SD	Total	Healthy Control Mean	SD	Total	Weight	Mean Difference IV, Fixed, 95% CI
Yehuda 2004	9.2	3.4	36	11.7	2.6	40	72.2%	-2.50 [-3.87, -1.13]
Yehuda 2006	9.57	3.2	14	10.2	3.2	19	27.8%	-0.63 [-2.84, 1.58]
**Total (95% CI)**			**50**			**59**	**100.0%**	**-1.98 [-3.14, -0.81]**
Heterogeneity: Chi² = 1.99, df = 1 (P = 0.16); I² = 50%								
Test for overall effect: Z = 3.33 (P = 0.0009)								

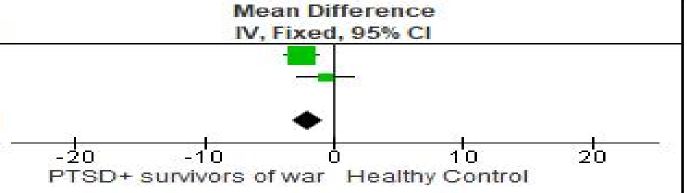

Figure 7 (d): CVLT- Delayed Recall (PTSD+ vs. Healthy Control)

Study or Subgroup	PTSD+ survivors of war Mean	SD	Total	Healthy Control Mean	SD	Total	Weight	Mean Difference IV, Fixed, 95% CI
Yehuda 2004	12.9	2.9	36	14.2	1.8	40	84.8%	-1.30 [-2.40, -0.20]
Yehuda 2006	9.57	3.8	14	10.47	3.7	19	15.2%	-0.90 [-3.49, 1.69]
**Total (95% CI)**			**50**			**59**	**100.0%**	**-1.24 [-2.25, -0.23]**
Heterogeneity: Chi² = 0.08, df = 1 (P = 0.78); I² = 0%								
Test for overall effect: Z = 2.40 (P = 0.02)								

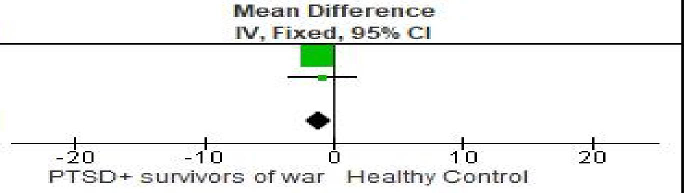

Figure 7 (e): CVLT- Short Recall (PTSD- vs. Healthy Control)

Study or Subgroup	Healthy Control Mean	SD	Total	PTSD- survivors of war Mean	SD	Total	Weight	Mean Difference IV, Fixed, 95% CI
Yehuda 2004	10.5	3.4	26	11.7	2.6	40	59.8%	-1.20 [-2.74, 0.34]
Yehuda 2006	10.23	2.2	13	10.2	3.2	19	40.2%	0.03 [-1.84, 1.90]
**Total (95% CI)**			**39**			**59**	**100.0%**	**-0.71 [-1.89, 0.48]**
Heterogeneity: Chi² = 0.99, df = 1 (P = 0.32); I² = 0%								
Test for overall effect: Z = 1.16 (P = 0.24)								

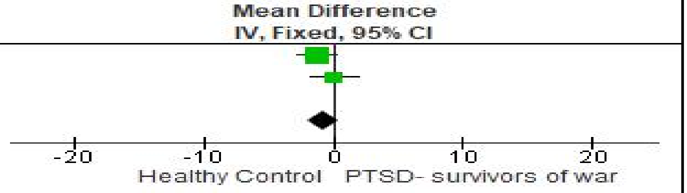

Figure 7 (f): CVLT- Delayed Recall (PTSD- vs. Healthy Control)

Study or Subgroup	Healthy Control Mean	SD	Total	PTSD- survivors of war Mean	SD	Total	Weight	Mean Difference IV, Fixed, 95% CI
Yehuda 2004	14	1.8	26	14.2	1.8	40	84.6%	-0.20 [-1.09, 0.69]
Yehuda 2006	10.78	2.3	13	10.47	3.7	19	15.4%	0.31 [-1.77, 2.39]
**Total (95% CI)**			**39**			**59**	**100.0%**	**-0.12 [-0.94, 0.70]**
Heterogeneity: Chi² = 0.20, df = 1 (P = 0.66); I² = 0%								
Test for overall effect: Z = 0.29 (P = 0.77)								

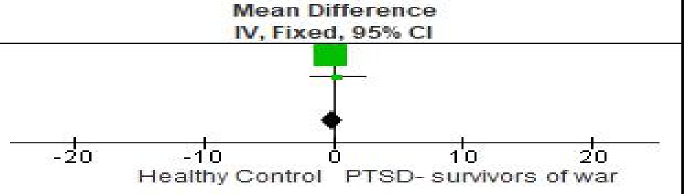

Learning and memory-Recognition memory

Figure 8 (a): CVLT Recognition memory (PTSD+ vs. PTSD-)

	PTSD+ survivors of war			PTSD- survivors of war				Mean Difference
Study or Subgroup	Mean	SD	Total	Mean	SD	Total	Weight	IV, Random, 95% CI
Freeman 2016	38.8	5.7	10	41.2	2.9	10	28.8%	-2.40 [-6.36, 1.56]
Hart 2008	39.2	3.4	7	41.2	2.9	11	48.7%	-2.00 [-5.05, 1.05]
Yehuda 2004	87.8	8.1	36	91.2	9.4	26	22.5%	-3.40 [-7.88, 1.08]
**Total (95% CI)**			**53**			**47**	**100.0%**	**-2.43 [-4.56, -0.30]**

Heterogeneity: Tau² = 0.00; Chi² = 0.26, df = 2 (P = 0.88); I² = 0%
Test for overall effect: Z = 2.24 (P = 0.03)

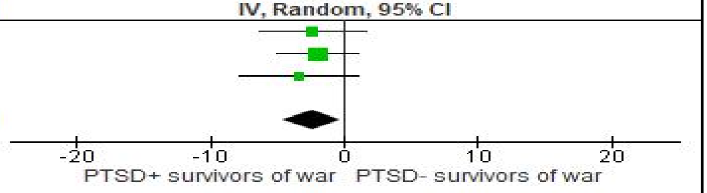

Figure 8 (b): CVLT Recognition memory (PTSD+ vs. Healthy Control)

	PTSD+ survivors of war			Healthy Controls				Mean Difference
Study or Subgroup	Mean	SD	Total	Mean	SD	Total	Weight	IV, Fixed, 95% CI
Freeman 2016	38.8	5.7	10	38.7	2.4	6	44.0%	0.10 [-3.92, 4.12]
Yehuda 2004	87.8	8.1	36	95.2	7.7	40	56.0%	-7.40 [-10.96, -3.84]
**Total (95% CI)**			**46**			**46**	**100.0%**	**-4.10 [-6.77, -1.43]**

Heterogeneity: Chi² = 7.49, df = 1 (P = 0.006); I² = 87%
Test for overall effect: Z = 3.01 (P = 0.003)

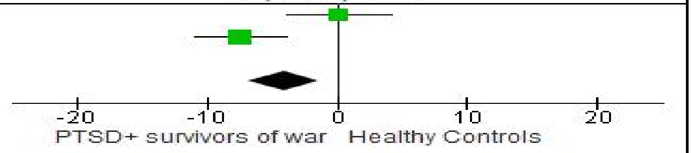

Figure 8 (c): CVLT Recognition memory (PTSD- vs. Healthy Control)

	Healthy Control			PTSD- survivors of war				Mean Difference
Study or Subgroup	Mean	SD	Total	Mean	SD	Total	Weight	IV, Fixed, 95% CI
Freeman 2016	38.7	2.4	6	41.2	2.9	11	10.7%	-2.50 [-5.07, 0.07]
Yehuda 2004	14.2	1.8	40	14	1.8	26	89.3%	0.20 [-0.69, 1.09]
**Total (95% CI)**			**46**			**37**	**100.0%**	**-0.09 [-0.93, 0.75]**

Heterogeneity: Chi² = 3.78, df = 1 (P = 0.05); I² = 74%
Test for overall effect: Z = 0.20 (P = 0.84)

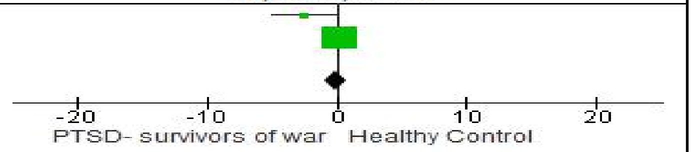

Verbal and non verbal Intelligence

Figure 9 (a): WAIS- Verbal IQ (PTSD+ vs. PTSD-)

	PTSD+ survivors of war			PTSD- survivors of war				Mean Difference
Study or Subgroup	Mean	SD	Total	Mean	SD	Total	Weight	IV, Random, 95% CI
Golier 2003	11	2.9	31	13.4	2.1	31	40.5%	-2.40 [-3.66, -1.14]
Yehuda 2004	11	2.8	36	13.7	2.5	26	36.5%	-2.70 [-4.03, -1.37]
Yehuda 2007	7.2	2.5	17	9.4	2.4	16	23.0%	-2.20 [-3.87, -0.53]
**Total (95% CI)**			**84**			**73**	**100.0%**	**-2.46 [-3.27, -1.66]**

Heterogeneity: Tau² = 0.00; Chi² = 0.23, df = 2 (P = 0.89); I² = 0%
Test for overall effect: Z = 6.02 (P < 0.00001)

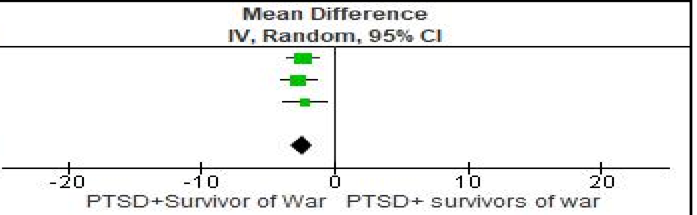

Figure 9 (b): WAIS- Verbal IQ (PTSD+ vs. Healthy Control)

	PTSD+ survivors of war			Healthy Control				Mean Difference
Study or Subgroup	Mean	SD	Total	Mean	SD	Total	Weight	IV, Random, 95% CI
Golier 2003	11	2.9	31	14.3	2.9	34	34.6%	-3.30 [-4.71, -1.89]
Yehuda 2004	11	2.8	36	14.2	3.2	40	37.9%	-3.20 [-4.55, -1.85]
Yehuda 2005	10.5	2.5	19	14.5	3	28	27.6%	-4.00 [-5.58, -2.42]
**Total (95% CI)**			**86**			**102**	**100.0%**	**-3.46 [-4.29, -2.63]**

Heterogeneity: Tau² = 0.00; Chi² = 0.64, df = 2 (P = 0.73); I² = 0%
Test for overall effect: Z = 8.16 (P < 0.00001)

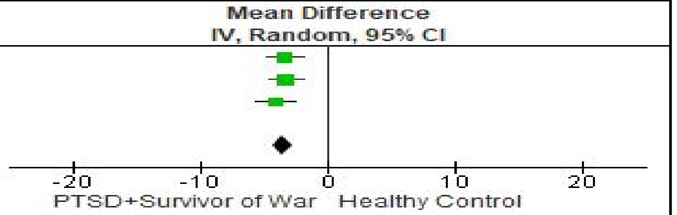

Figure 9 (c): WAIS- Verbal IQ (PTSD- vs. Healthy Control)

	Healthy Control			PTSD- survivors of war				Mean Difference
Study or Subgroup	Mean	SD	Total	Mean	SD	Total	Weight	IV, Random, 95% CI
Golier 2003	13.4	2.1	16	14.3	2.9	34	34.1%	-0.90 [-2.32, 0.52]
Yehuda 2004	13.7	2.5	26	14.2	3.2	40	36.0%	-0.50 [-1.88, 0.88]
Yehuda 2005	13.7	2.1	16	14.5	3	28	29.9%	-0.80 [-2.31, 0.71]
**Total (95% CI)**			**58**			**102**	**100.0%**	**-0.73 [-1.55, 0.10]**

Heterogeneity: Tau² = 0.00; Chi² = 0.17, df = 2 (P = 0.92); I² = 0%
Test for overall effect: Z = 1.72 (P = 0.09)

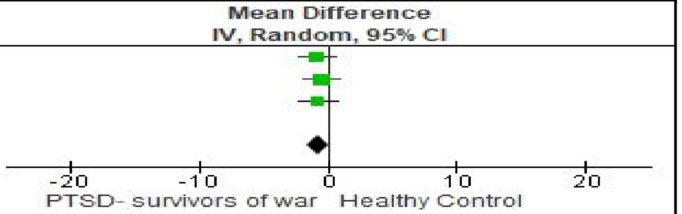

Figure 9 (a): WAIS- Non verbal IQ (PTSD+ vs. PTSD-)

	PTSD+ survivors of war			PTSD- survivors of war				Mean Difference
Study or Subgroup	Mean	SD	Total	Mean	SD	Total	Weight	IV, Fixed, 95% CI
Yehuda 2004	9.7	3.3	36	11.3	2.3	26	71.7%	-1.60 [-2.99, -0.21]
Yehuda 2007	10.3	3.5	17	10.7	3	16	28.3%	-0.40 [-2.62, 1.82]
**Total (95% CI)**			**53**			**42**	**100.0%**	**-1.26 [-2.44, -0.08]**

Heterogeneity: Chi² = 0.80, df = 1 (P = 0.37); I² = 0%
Test for overall effect: Z = 2.09 (P = 0.04)

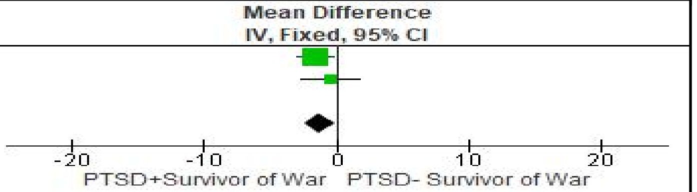

Figure 9 (b): WAIS- Non verbal IQ (PTSD+ vs. Healthy Control)

	PTSD+ survivors of war			Healthy Control				Mean Difference
Study or Subgroup	Mean	SD	Total	Mean	SD	Total	Weight	IV, Fixed, 95% CI
Yehuda 2004	9.7	3.3	36	11.3	2.6	40	54.4%	-1.60 [-2.95, -0.25]
Yehuda 2005	8.6	3.7	19	10.6	2.8	28	25.6%	-2.00 [-3.96, -0.04]
Yehuda 2007	10.3	3.5	17	10.7	3	16	20.0%	-0.40 [-2.62, 1.82]
**Total (95% CI)**			**72**			**84**	**100.0%**	**-1.46 [-2.46, -0.47]**

Heterogeneity: Chi² = 1.21, df = 2 (P = 0.55); I² = 0%
Test for overall effect: Z = 2.89 (P = 0.004)

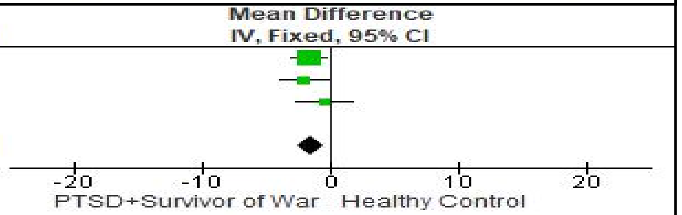

Figure 9 (c): WAIS- Non verbal IQ (PTSD- vs. Healthy Control)

	Healthy Control			PTSD- survivors of war				Mean Difference
Study or Subgroup	Mean	SD	Total	Mean	SD	Total	Weight	IV, Fixed, 95% CI
Yehuda 2004	11.3	2.6	40	11.3	2.3	26	57.6%	0.00 [-1.20, 1.20]
Yehuda 2005	10.6	2.8	28	10.9	1.9	16	42.4%	-0.30 [-1.69, 1.09]
**Total (95% CI)**			**68**			**42**	**100.0%**	**-0.13 [-1.03, 0.78]**

Heterogeneity: Chi² = 0.10, df = 1 (P = 0.75); I² = 0%
Test for overall effect: Z = 0.27 (P = 0.78)

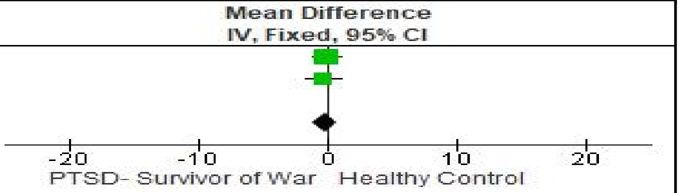

AIMS Press

© 2021 the Author(s), licensee AIMS Press. This is an open access article distributed under the terms of the Creative Commons Attribution License (http://creativecommons.org/licenses/by/4.0)
